# MKRN1 promotes colorectal cancer metastasis by activating the TGF-β signalling pathway through SNIP1 protein degradation

**DOI:** 10.1186/s13046-023-02788-w

**Published:** 2023-08-24

**Authors:** Yi Zhang, Qin-shan Li, Hong-lin Liu, Hong-ting Tang, Han-lin Yang, Dao-qiu Wu, Yu-ying Huang, Li-cheng Li, Li-hong Liu, Meng-xing Li

**Affiliations:** 1 Guizhou Prenatal Diagnosis Center, Affiliated Hospital of Guizhou Medical University, Guiyang, 550004 Guizhou People’s Republic of China; 2 Department of Clinical Biochemistry, School of Medical Laboratory Science, Guizhou Medical University, Guizhou Guiyang, 550004 People’s Republic of China; 3 Institute of Clinical Medical Sciences, China-Japan Friendship Hospital, Beijing, 100000 People’s Republic of China; 4 Clinical Medical College, Guizhou Medical University, Guizhou Guiyang, 550004 People’s Republic of China; 5 Department of HematologyGuizhou Province Laboratory of Hematopoietic Stem Cell Transplantation Centre, Affiliated Hospital of Guizhou Medical University, Guizhou Province Institute of Hematology, Guizhou Guiyang, People’s Republic of China; 6 Department of Pharmacy, China-Japan Friendship Hospital, Beijing, 100029 People’s Republic of China; 7 Department of Pathophysiology, Guizhou Medical University, Guizhou Guiyang, 550004 People’s Republic of China

**Keywords:** Colorectal cancer, *MKRN1*, Metastasis, SNIP1, TGF-β signalling

## Abstract

**Background:**

The Makorin ring finger protein 1 (*MKRN1*) gene, also called RNF61, is located on the long arm of chromosome 7 and is a member of the RING finger protein family. The E3 ubiquitin ligase MKRN1 is closely linked to tumour development, but the exact mechanism needs to be elucidated. In this study, we aimed to investigate the specific mechanism and role of *MKRN1* in colorectal cancer (CRC) development.

**Methods:**

*MKRN1* expression in CRC was analysed using the Cancer Cell Line Encyclopaedia and the Cancer Genome Atlas (TCGA) databases. Rectal tumour tissues were frozen to explore the MKRN1 expression in CRC and its clinical significance. The impact of *MKRN1* on CRC cell proliferation and migration was observed using CCK8, colony formation, wound healing, and transwell assays. A combination of MKRN1 quantitative proteomics, ubiquitination modification omics analysis, and a string of in vitro and in vivo experiments revealed the potential mechanisms by which *MKRN1* regulates CRC metastasis.

**Results:**

*MKRN1* expression was significantly elevated in CRC tissues compared to paracancerous tissues and was positively linked with prognosis (*P* < 0.01). *MKRN1* downregulation inhibits CRC cell proliferation, migration, and invasion. Conversely, *MKRN1* overexpression promotes the proliferation, migration, and invasion of CRC cells. Mechanistically, *MKRN1* induces epithelial-mesenchymal transition (EMT) in CRC cells via ubiquitination and degradation of Smad nuclear-interacting protein 1 (SNIP1). Furthermore, SNIP1 inhibits transforming growth factor-β (TGF-β) signalling, and *MKRN1* promotes TGF-β signalling by degrading SNIP1 to induce EMT in CRC cells. Finally, using conditional knockout mice, intestinal lesions and metastatic liver microlesions were greatly reduced in the intestinal knockout *MKRN1* group compared to that in the control group.

**Conclusions:**

High *MKRN1* levels promote TGF-β signalling through ubiquitination and degradation of SNIP1, thereby facilitating CRC metastasis, and supporting *MKRN1* as a CRC pro-cancer factor. The MKRN1/SNIP1/TGF-β axis may be a potential therapeutic target in CRC.

**Supplementary Information:**

The online version contains supplementary material available at 10.1186/s13046-023-02788-w.

## Background

Colorectal cancer (CRC) is the world's third most common cancer [1]. The yearly global incidence exceeds one million, and fatality rates in developed countries can reach 33% [2]. The population of patients with this disease is gradually becoming younger [3]. Liver metastasis from CRC is the most substantial factor affecting survival rates. Approximately one-quarter of patients already have metastases at the time of diagnosis, which directly contributes to the overall poor prognosis and high mortality rate of patients with CRC [4]. Accordingly, understanding the molecular pathways underlying metastatic development in CRC and identification of new biomarkers for use in diagnosing and treating patients with CRC is critical.

CRC occurrence and development are closely related to the abnormal regulation of ubiquitin ligases [5]. The Makorin ring finger protein 1 (*MKRN1*) gene is localized on the long arm of chromosome 7. It is highly conserved among different species [6]. MKRN1 contains a functional ring-finger structural domain of E3 ubiquitin ligase [7]. RINGE3 ubiquitin ligases regulate essential cellular biological processes of cell proliferation, apoptosis, and epithelial-mesenchymal transition (EMT) as oncogenes or tumour suppressors, depending on the cell environment, enzyme expression level, and target substrates [8, 9]. MKRN1 ubiquitin ligase plays a role in the development of various tumours, mediating the ubiquitination of different substrate proteins. These substrate proteins include p14ARF, human telomerase reverse transcriptase (hTERT), Fas-associated protein with death domain (FADD), phosphatase and tensin homologue (PTEN), p53, and p21, all of which have been implicated in tumourigenesis following ubiquitination [10–14]. Thus, down-regulation of p14ARF MKRN1-mediated degradation results in the inhibition of tumour cell senescence [10]. Similarly, tHERT and FADD degradation through increased MKRN1 activity leads, respectively, to reduced telomerase activity (and reduced telomere length) and reduced tumour cell apoptosis and necrosis [11, 12]. Moreover, ubiquitination of PTEN accelerates tumourigenesis [13], whereas MKRN1 activity against p53 and p21 interferes with the cell cycle and apoptosis [14]. Overall, these findings support a potential tumour-promoting function of *MKRN1* in cancer initiation and maintenance. However, its role and mechanisms in colorectal tumour development are unclear and need to be investigated in depth.

Smad nuclear-interacting protein 1 (SNIP1) is a novel interacting factor of the Smad protein family that is expressed in different cell types and is involved in cell proliferation, apoptosis, and differentiation [15]. SNIP1 competes with RelA/p65, a subunit of NF-κB, for binding to p300, inhibiting the NF-κB signalling pathway [16]. SNIP1 inhibits TGF-β-mediated lung cancer cell migration [17]. In osteosarcoma cells, SNIP1 downregulation reduces p53 expression under UV treatment; SNIP1 is also involved in the ataxia telangiectasia-mutated and Rad3-related protein-mediated tumour suppressor function of the p14 gene [18]. In addition, SNIP1 inhibits TGF-β pathway activation [19]. However, the role of SNIP1 in CRC needs to be urgently elucidated.

In the current investigation, we identified MKRN1 expression in CRC and studied its potential prognostic value. We also revealed the mechanism by which *MKRN1* causes invasive metastasis in CRC. We discovered that patients with CRC who had high MKRN1 expression have poor prognoses. *MKRN1* deletion in CRC cells reduces EMT-mediated metastasis in vivo and in vitro. Mechanistically, *MKRN1* promotes TGF-β signalling by inhibiting SNIP1 to induce EMT and metastasis in CRC cells. Overall, this study confirms for the first time that *MKRN1* is a novel metastasis-promoting factor in CRC, and for the first time, SNIP1 was found to be degraded as a ubiquitinated substrate of MKRN1 in CRC.

## Methods

### Clinical specimens of patients

Histopathological sections of four cases of colitis and 16 patients with CRC who underwent radical colorectal surgery were randomly selected at the Affiliated Hospital of Guizhou Medical University (Guiyang, Guizhou) during September to December 2019. Tumour tissues and matched normal fresh tissues were collected from three CRC patients. The Ethical Review Committee of Guizhou Medical University authorized this investigation, and all patients provided informed permission prior to the study (2019–055).

### Immunohistochemistry, western blotting, and quantitative real-time PCR analysis

Additional file 1 contains information on the immunohistochemistry (IHC). Image-J software was used to calculate integrated optical density (IOD), and the results are presented as average optical density values (AOD), calculated as AOD = IOD/area. ImageJ software was used to analyse the relevant protein expression by western blotting (WB). Information on the primary antibodies is listed in Additional file 2. The SYBR Green real-time PCR master mix was used to extract RNA from CRC cell lines (Takara, Japan). PCR primers can be found in Additional file 3.

### Cell lines and transfection

Normal human colonic fibroblasts CCD-18Co were purchased from the American Type Culture Collection, and human CRC cell lines HCT116, HT29, HCT15, and RKO were purchased from the Cell Centre of the Institute of Basic Medical Sciences, Chinese Academy of Medical Sciences. All cell lines were grown in full Dulbecco's modified Eagle’s medium (DMEM) with 10% foetal bovine serum and 1% penicillin/streptomycin combination at 37 ℃ and 5% CO_2_ in a cell incubator.

The HCT116 cells were transfected with the *MKRN1* short hairpin RNA (shRNA) lentiviral vector constructed during this study and the Tet-pTripz-puro plasmid purchased from Thermo Fisher (Waltham, MA, USA). Virus packaging plasmids psPAX2 and pMD2.G were obtained from the Kunming Institute of Zoology, Chinese Academy of Sciences. HCT15 cells, transfected with *MKRN1* or *SNIP1* cDNA lentiviral vectors or the corresponding control vector, were purchased from Sino Biological (Beijing, China). Guangzhou IGE Biotechnology Ltd synthesised the *MKRN1*(H307E) and *SNIP1* shRNAs.

The *MKRN1* shRNA target sequence can be found in Additional file 3. The target cells were screened by infecting the corresponding cells with the viral solution and incubating them in a medium containing puromycin.

### Cell proliferation assay and cell migration and invasion

In the cell proliferation assay, 1 × 10^3^ cells were cultured in 96-well plates, and the optical density was assessed at 0, 1, 3, 5, and 7 days using the cell counting Kit-8 (CCK-8; Dojindo, Japan). To count the number of colonies generated, 500 cells were cultured in 6-well plates for 12 days (fresh medium supplied every 3–4 days) and stained with 1% crystal violet (Solarbio, Beijing, China). CRC cells were cultured overnight in serum-free DMEM for migration analysis (well size, 8 µm; Corning Costar, Corning, NY, USA) and Matrigel (Corning Costar, Corning, NY, USA) to detect cell invasion. The following day, in the upper chamber, starved cells were inoculated, and 600 µL of culture media containing 10% foetal bovine serum added in the bottom chamber. Following 48 h at 37 ℃, the chambers were fixed with 4% paraformaldehyde (Leagene Biotechnology, Beijing, China) and stained for 10 min with crystal violet. After removing the excess dye with phosphate-buffered saline, the cells in the intracellular layer were examined under a microscope after being cleaned with a cotton swab. Cell migration assays were performed by adding 5 × 10^4^ or 4 × 10^4^ of HCT116 and HCT15 cells, respectively, to the upper chamber of a transwell, with measurement after 48 h. Matrigel gel was prepared for the invasion assay in a ratio of 1:8 in the culture medium. Then, 60 µL of the mixture was added to each well, and 600 µL of culture medium containing 20% foetal bovine serum was added to the lower chamber. For the assay, 6 × 10^4^ cells (HCT116) or 5 × 10^4^ cells (HCT15) were seeded into the upper chamber. The remaining steps were the same as for the migration experiment. Three separate experiments were performed for each group.

### Co-immunoprecipitation

Lysis buffer was prepared at an NP40: protease inhibitor ratio of 50:1. Then, 600 µL of the lysis buffer was added to the cell precipitate and placed on a low-speed rotary shaker at 4 ℃ for 30 min. After centrifugation at 13,000 × *g* at 4 ℃ for 20 min, the supernatant was transferred to a new tube. Then, 100 µL was used as the input control, and 20 µL of protein A/G agarose beads were added to the remainder and incubated for 30 min at 4 ℃ with slow shaking. After centrifuging the mixture at 3000 rpm for 10 min, the supernatant was collected and deposited in a fresh Eppendorf tube. Cell protein was quantifying using the BCA protein assay; the antibody against the target protein was added to the immunoprecipitation (IP) group. The IgG antibody with the same properties as the target antibody was then added to the IgG group, followed by overnight incubation with the antibody and protein. Then, 35 µL of protein A/G agarose beads were added to the IP and IgG groups and incubated overnight at 4 ℃ with slow shaking. The next day, the agarose beads were washed three times with RIPA buffer before adding 70 µL of 1 × loading buffer, and the sample was boiled for 10 min at 100 ℃ in a metal bath. Sodium dodecyl-sulphate polyacrylamide gel electrophoresis was performed on 8% separating gels. Finally, the immunoblots were probed with the appropriate antibodies, and electrochemiluminescence assays were performed.

### Quantitative proteomics and ubiquitination modifications

HT29, HCT116-control, and sh-*MKRN1* cells were collected for proteomic studies and ubiquitination modification histology. The study was carried out by Jing jie PTM BioLab (Hangzhou) Co. Inc. The specific methods are described in Additional file 4.

### Ubiquitination analysis

Ubiquitination was measured in HCT116-Control, HCT116-sh1-*MKRN1*, HCT15-Vector, and HCT15-OE-*MKRN1* cells treated with MG132 (10 µmol, MCE, San Rafael, CA, USA) for 6 h. Both cell lines were then subjected to IP followed by WB. The half-life of SNIP1 was determined using the cycloheximide (CHX) chase assay approach. HCT116-Control, HCT116-sh1-*MKRN1*, HCT15-Vector, and HCT15-OE-*MKRN1* cells were treated with CHX (50 µg/mL; Solarbio, Beijing, China) for 2, 4, and 6 h, and SNIP1 was detected using WB.

### Animal study

Mutant Adenomatous polyposis coli (Apc) *MKRN1* conditional knockout mice were purchased from Cyagen Biologicals, Ltd., and were categorized into groups as follows: Apc [MU/ +], Mkrn1 [+ / + , pVillin-Cre], Apc [MU/ +], and Mkrn1 [flox/flox, pVillin-Cre]. Mice were grown in specific-pathogen-free class animal houses and were euthanized after 33 weeks.

### Gene set enrichment analysis (GSEA)

We downloaded gene sets from the Reactome pathway database in the Human MSigDB Collections. After ID conversion of the molecular list of the differential expressed genes in the SNIP1 high and low expression groups, GSEA was performed using the clusterProfiler [4.4.4] package Of R (version 4.2.1).

### Statistical analysis

SPSS Statistics 26.0 software was used for statistical analysis. All experiments were repeated three times. Experimental results are presented as the mean ± standard deviation. *t*-test was used to compare the two groups. *P* < 0.05 indicated statistical significance.

## Results

### *MKRN1* expression is upregulated in CRC and associated with poor prognosis

**Fig. 1:**
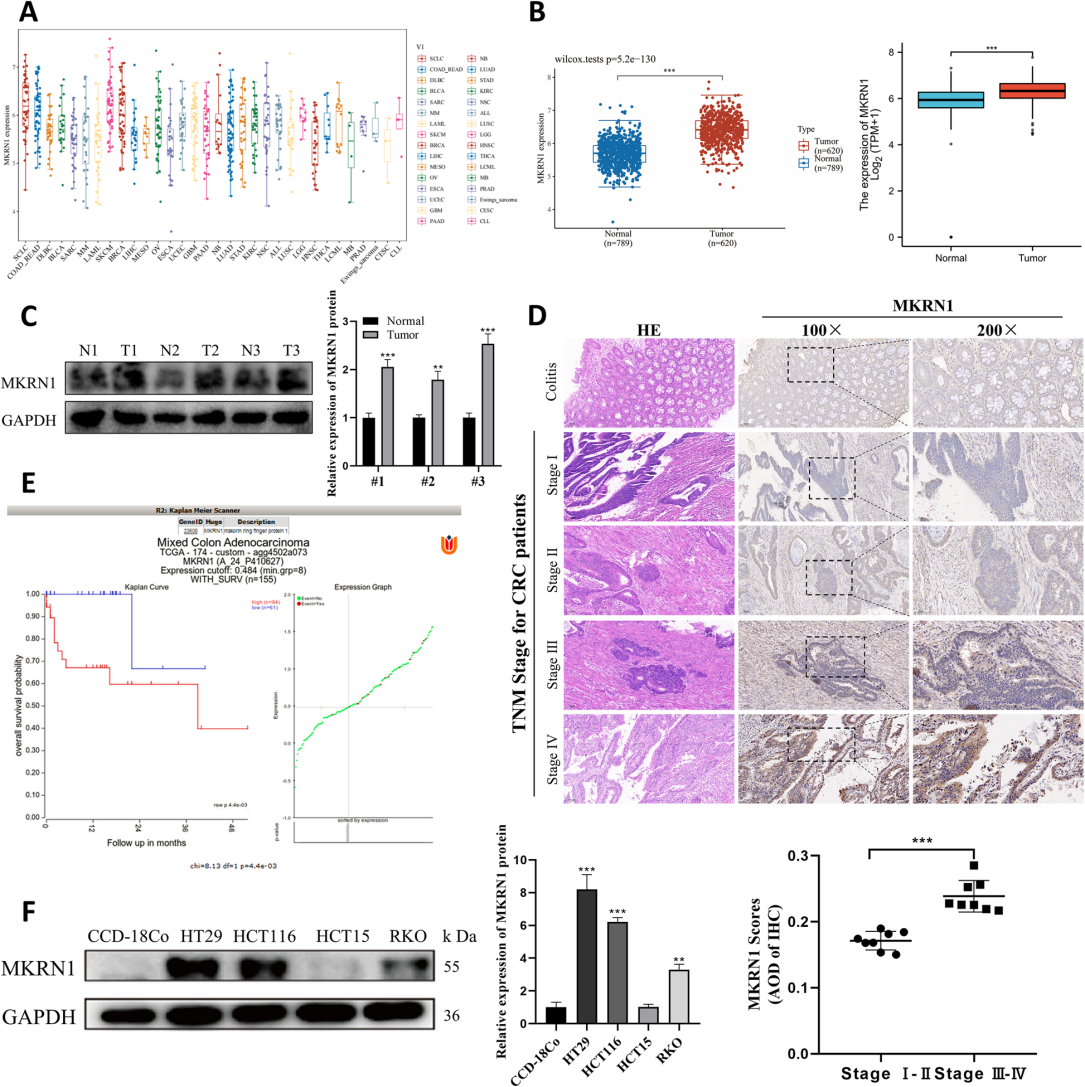
*MKRN1* is highly expressed and associated with poor prognosis in patients with CRC. **A** The expression distribution of mRNA in different tumour cell lines. **B** The distribution of *MKRN1* expression in tumour and normal tissues. **C** MKRN1 expression in CRC tumour and adjacent non-tumour tissues was verified by WB analysis. **D** Immunohistochemistry detection of MKRN1 expression in tissue sections of patients with CRC and colitis showing typical photographs (scale bars are 100 and 50 µm, respectively). **E** The Kaplan–Meier Plotter in the R2 Genomics Analysis Platform was used to draw the overall survival curve. **F** WB analysis of MKRN1 expression levels in CRC cells (HT29, HCT116, HCT15, and RKO) and normal human colonic fibroblasts (CCD-18Co). ** *P* < 0.01, *** *P* < 0.001

To explore MKRN1 expression in tumours, the Cancer Cell Line Encyclopaedia dataset (https://portals.broadinstitute.org/ccle) was used to obtain the cell line expression matrix of colorectal tumours. *MKRN1* was highly expressed in CRC (Fig. 1A). The Cancer Genome Atlas (TCGA) dataset (https://portal.gdc.com) was also used to retrieve RNA-sequencing expression profiles and clinical information for 620 colorectal tumours. *MKRN1* expression levels in CRC and normal tissues were detected using TCGA in conjunction with the GTEx database, which showed that *MKRN1* was highly expressed in CRC compared with normal tissues (Fig. 1B). In three pairs of CRC and adjacent non-tumour tissues, we also measured MKRN1 protein levels, and discovered that its expression was more prominent in the CRC tissues than in the adjoining cancer tissues (Fig. 1C).

IHC staining of tissue sections from patients with CRC indicated that MKRN1 expression was more prominent in CRC tissues than in patients with colitis. MKRN1 expression correlated with the tumour, node, and metastasis (TNM) stage of the tumour, and was significantly higher in patients with stages III–IV than in patients with stages I–II cancer (Fig. 1D, *P* < 0.001). Additionally, the R2 Genome Analysis Platform (https://hgserver1.amc.nl/cgi-bin/r2/main.cgi) study revealed that patients with CRC with high *MKRN1* expression levels had significantly shorter overall survival than those with low *MKRN1* expression levels (Fig. 1E, *P* < 0.001).

To further understand the role of MKRN1 in CRC formation, we examined the protein expression of MKRN1 in four CRC cell lines and in normal human colonic CCD-18Co fibroblasts using WB. Compared with CCD-18Co cells, the CRC cell lines showed higher MKRN1 protein expression in HT29, HCT116, and RKO cells and lower expression in HCT15 cells (Fig. 1F). These findings suggest that MKRN1 is substantially elevated in CRC tissues and is linked with poor prognosis in patients with CRC.

### MKRN1 promotes CRC cell proliferation, migration, and invasion and induces EMT

**Fig. 2:**
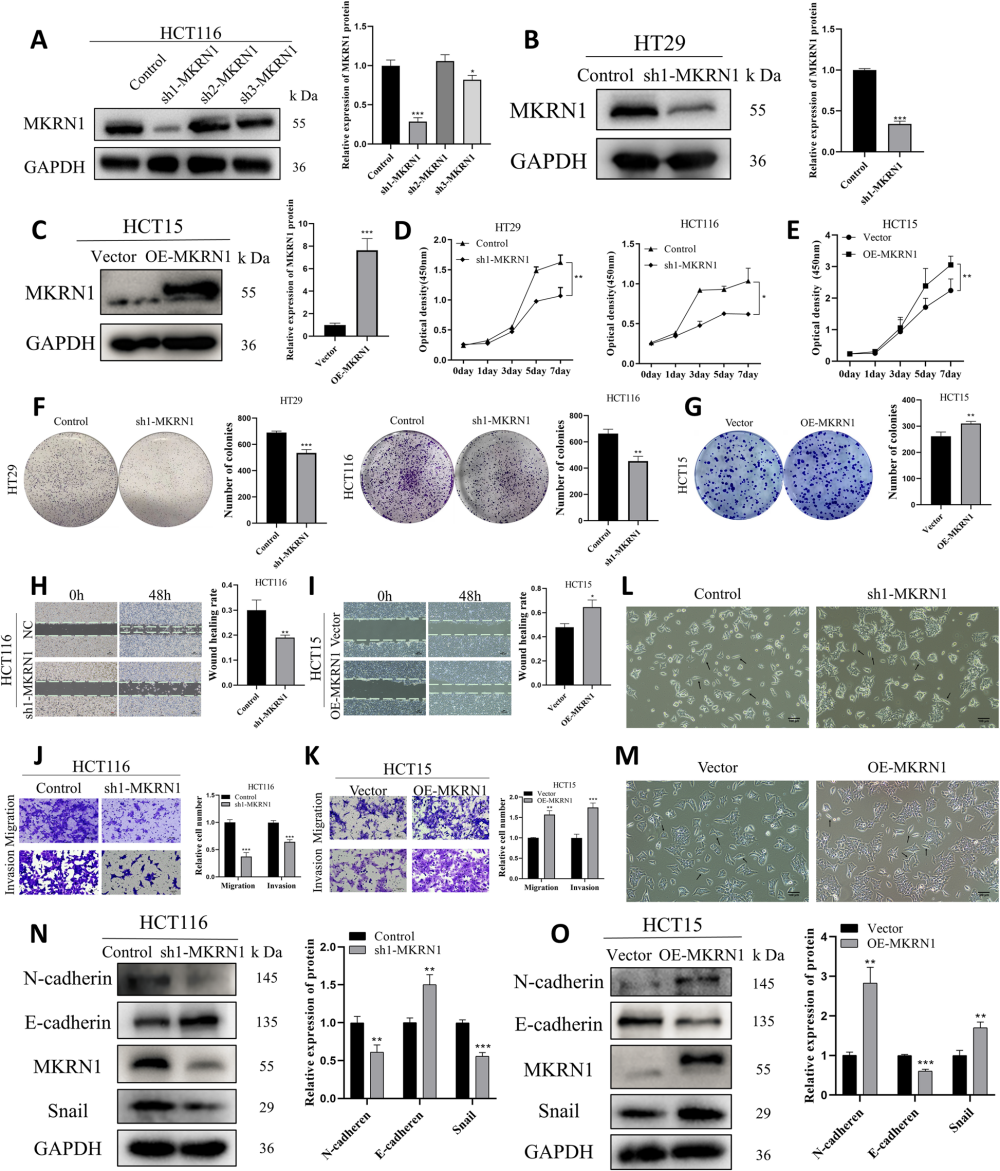
*MKRN1* has a role in CRC cell proliferation, migration, and invasion. **A**–**C** WB analysis of *MKRN1* transfection rates in HCT116, HT29, and HCT15 cells. **D**, **E** CCK-8 assay for CRC cell viability. **F**, **G** Colony formation assay to detect CRC cell proliferation. **H**, **I** Migration ability of CRC cells with different *MKRN1* expression levels detected using a wound healing assay (Scale bar: 100 µm). **G**-**K** Transwell assays for migration and invasion of *MKRN1* knockdown and overexpressing cells (Scale bar: 50 µm). **L**, **M** Microscopic observation of the morphology of the cell lines HCT116 (Control, sh1-MKRN1) and HCT15 (Vector, OE-MKRN1) (Scale bar: 100 µm). **N**, **O** Comparison of epithelial and mesenchymal marker expression following knockdown and *MKRN1* overexpression. * *P* < 0.05, ** *P* < 0.01, *** *P* < 0.001

Based on the MKRN1 expression in CRC cells, we selected HCT116 and HT29 cells with high MKRN1 expression for knockdown and HCT15 cells with low MKRN1 expression for overexpression. We designed three shRNAs for *MKRN1*, and WB showed that sequence 1 effectively knocked down MKRN1, whereas sequences 2 and 3 were ineffective (Fig. 2A). HT29 cells were successfully knocked down using sequence 1 (Fig. 2B). The effect of MKRN1 overexpression in HCT15 cells was detected by WB (Fig. 2C).

Cell viability was examined using the CCK8 assay; this showed that the viability of HCT116 and HT29 cells decreased after *MKRN1* knockdown (Fig. 2D). In contrast, the cell viability of HCT15 increased after *MKRN1* overexpression (Fig. 2E). The colony formation assay results showed that after *MKRN1* knockdown, HCT116 and HT29 cells formed fewer and smaller colonies (Fig. 2F), and after *MKRN1* overexpression, HCT15 cells formed more and larger colonies (Fig. 2G). In summary, *MKRN1* promotes CRC cell proliferation.

We chose highly invasive CRC cells HCT116 to explore whether *MKRN1* promotes CRC metastasis [20]. The wound healing test was used to evaluate the capacity of cells to migrate, which showed *MKRN1* knockdown in HCT116 cells inhibited the scratch healing rate of CRC cells (Fig. 2H). In HCT15 cells, *MKRN1* overexpression promoted the rate of wound healing in CRC cells (Fig. 2I). Using a transwell assay, the migration and invasive ability of HCT116 and HCT15 cells was determined. The ability of CRC cells to migrate and invade was suppressed when *MKRN1* was knocked down in HCT116 cells (Fig. 2J), while it was boosted when *MKRN1* was overexpressed in HCT15 cells (Fig. 2K).

EMT plays a critical and complex role in tumour invasion and metastasis. Using microscopy, we found that *MKRN1* knockdown changed HCT116 cells from their original fibroblast morphology (spindle-shaped) to epithelial morphology (tightly bound). In contrast, *MKRN1* overexpression changed HCT15 from its original epithelial to fibroblast morphology (Fig. 2L, M). At the protein level, *MKRN1* knockdown boosted E-cadherin expression while decreasing N-cadherin and Snail levels, consistent with the morphological alterations. *MKRN1* overexpression, on the other hand, enhanced N-cadherin and Snail expression while decreasing E-cadherin expression (Fig. 2N, O). In conclusion, these data suggest that *MKRN1* positively regulates EMT and CRC cell migration and invasion.

### MKRN1 interacts with SNIP1

**Fig. 3:**
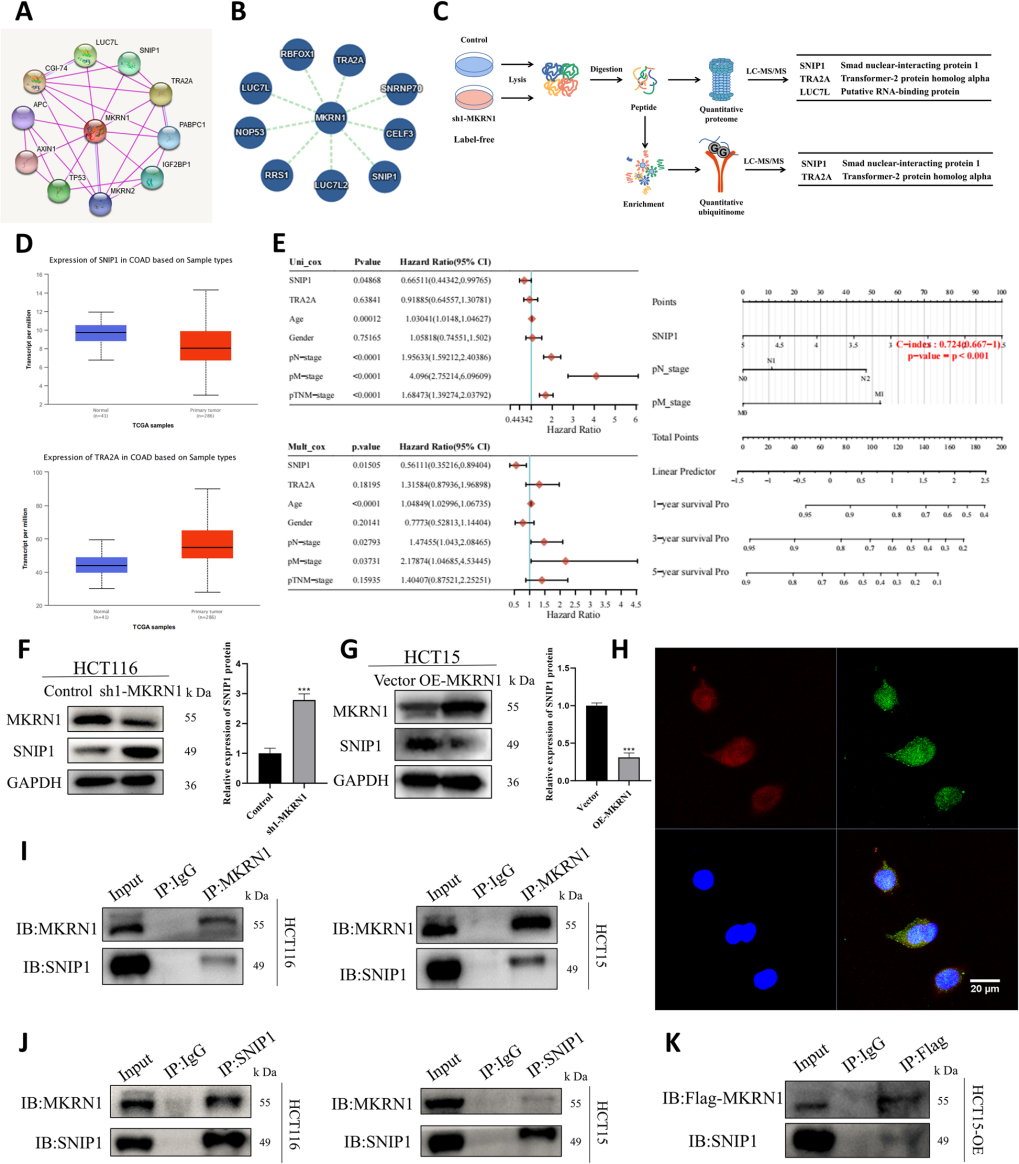
MKRN1 and SNIP1 interaction. **A** Prediction of protein interaction with MKRN1 using the STRING database. **B** Prediction of protein interaction with MKRN1 using the IntAct database. **C** The experimental process of MKRN1 quantitative proteomics and ubiquitination modification omics. **D** Expression distribution of *SNIP1* (upper) and *TRA2A* (lower) in colorectal tumour and normal tissues. **E** Univariate and multifactorial Cox analyses of *P*-values, hazard rate, and confidence intervals for gene expression and clinical characteristics. **F**, **G** WB analysis showing that MKRN1 expression level affects SNIP1 protein expression. **H** Confocal microscopy showing MKRN1 and SNIP1 localisation (Scale bar: 20 µm). **I**, **J** Forward and reverse validation of MKRN1 interactions with SNIP1 in HCT116 and HCT15 cells using co-immunoprecipitation (Co-IP) and WB. **K** Validation of exogenous MKRN1 interaction with SNIP1 in HCT15 cells using Co-IP and WB. *** *P* < 0.001

To investigate the potential mechanism through which *MKRN1* functions, we searched the STRING and the IntAct databases for proteins that may interact with MKRN1 (Fig. 3A, B). Three potential proteins were identified: SNIP1, TRA2A, and LUC7L. Subsequently, we conducted proteomic and ubiquitination modification omics studies, which revealed that MKRN1 may interact with SNIP1 and TRA2A (Fig. 3C). To discover the functions of SNIP1 and TRA2A in CRC, we analysed TCGA database and found that SNIP1 showed low expression in CRC compared to normal controls (Fig. 3D upper), but TRA2A was highly expressed in CRC (Fig. 3D lower). With TCGA dataset, we obtained the RNA-sequencing expression patterns and associated clinical data for 620 colorectal tumours. Using univariate and multivariate Cox regression analysis, it was shown that SNIP1 could be an independent prognostic factor for CRC, whereas TRA2A was rejected as a potential prognostic indicator (Fig. 3E).

Interestingly, one study discovered a considerable drop in SNIP1 expression in individuals with inflammatory bowel disease and intraepidermal carcinoma in a mouse model of colitis [21]. *TRA2A* is an oncogene highly expressed in various tumours [22–25]. Hence, we examined the effect of *MKRN1* alteration on SNIP1 expression levels by WB, which showed that *MKRN1* knockdown resulted in upregulation of SNIP1 expression, and *MKRN1* overexpression resulted in downregulation of SNIP1 expression (Fig. 3F, G), thus revealing a negative correlation between MKRN1 and SNIP1 expression levels. As SNIP1 protein expression was significantly downregulated in cells with high *MKRN1* expression, we sought to elucidate the relationship between MKRN1 and SNIP1 proteins in CRC cells. Therefore, we examined the spatial distribution of MKRN1 and SNIP1 by confocal imaging, which showed that MKRN1 expression (red) partially overlapped with that of SNIP1 (green), indicating that both were co-localised in the cytoplasm and nucleus (Fig. 3H). Co-immunoprecipitation (Co-IP) combined with WB verified that endogenous MKRN1 and SNIP1 formed a complex in HCT116 and HCT15 cells (Fig. 3I, J), whereas exogenous MKRN1 and endogenous SNIP1 could still create a complex (Fig. 3K). These results suggested that MKRN1 interacts with SNIP1.

### MKRN1 induces SNIP1 proteasomal degradation and promotes SNIP1 ubiquitination

**Fig. 4:**
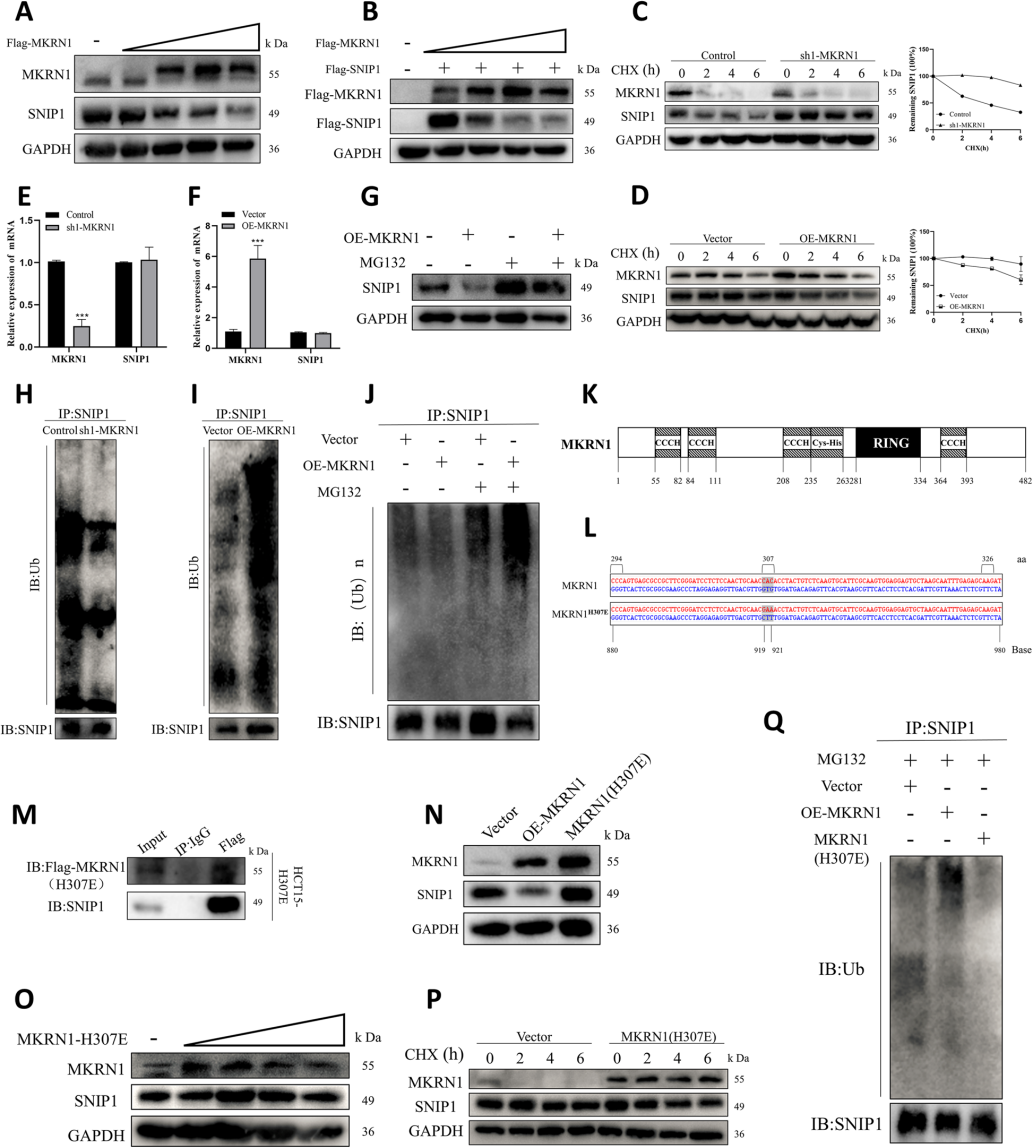
Degradation of ubiquitinated SNIP1 by MKRN1 requires E3 ligase activity. **A** Degradation of SNIP1 by MKRN1. Expression vectors for Flag-*MKRN1* (0, 1, 2, 4, and 6 µg) were introduced into HCT15 cells; steady-state expression levels of SNIP1 and MKRN1 were determined by WB. **B** Expression vectors containing Flag-*SNIP1* (1 µg) and Flag-*MKRN1* (1, 2, 4, and 6 µg) were introduced into HCT15 cells; steady-state expression levels of SNIP1 and MKRN1 were detected using WB. **C**, **D** MKRN1 affects half-life of SNIP1. HCT116 cells (Control and sh1-MKRN1) and HCT15 cells (vector and OE-MKRN1) were treated with 50 µg/mL cycloheximide and analysed by WB. **E**, **F** Effects of *MKRN1* on *SNIP1* mRNA by quantitative real-time PCR. **G** Proteasome-dependent degradation of SNIP1 by MKRN1 in HCT15 cells overexpressing *MKRN1* and cellular SNIP1 expression after 6 h treatment with 10 µM MG132. **H**, **I** MKRN1 mediates SNIP1 ubiquitination. In HCT116 and HCT15 cells, SNIP1 ubiquitination levels after *MKRN1* knockdown and overexpression were detected by WB. **J** In HCT15 cells, the SNIP1 ubiquitination level was measured by WB after a 6-h treatment with 10 µM MG132. **K** Schematic diagram of the *MKRN1* structural domain. **L** Construction of the E3 ligase mutant H307E of *MKRN1*. **M** Validation of MKRN1 (H307E) interactions with SNIP1 using co-immunoprecipitation and WB in HCT15 (H307E) cells. **N** WB analysis of the effect of the *MKRN1* (H307E) mutant on SNIP1 degradation. **O** SNIP1 degradation by *MKRN1* (H307E). Expression vectors for *MKRN1* (H307E) (0, 1, 2, 4, and 6 µg) were introduced into HCT15 cells; WB detected steady-state expression levels of SNIP1 and MKRN1. **P** Effect of *MKRN1* (H307E) on half-life of SNIP1. HCT15 cells (vector and *MKRN1* (H307E)) were treated with 50 µg/mL cycloheximide and analysed by WB. **Q** In HCT15 cells (vector, OE-*MKRN1*, *MKRN1* (H307E)), SNIP1 ubiquitination levels were detected by WB after a 6-h treatment with 10 µM MG132

Based on the finding that MKRN1 deletion stabilises SNIP1, we next examined the stabilisation level of SNIP1 in HCT15 cells with increasing amounts of exogenous MKRN1. As predicted, endogenous SNIP1 levels gradually dropped in the presence of exogenous MKRN1 (Fig. 4A). Simultaneously, the stable exogenous SNIP1 level gradually decreased as the exogenous MKRN1 level increased (Fig. 4B). To determine the cause of this SNIP1 destabilisation, we treated CRC cells with the protein synthesis inhibitor CHX and measured the half-life of SNIP1. Kinetic studies of SNIP1 degradation in the presence of CHX showed that MKRN1 knockdown increased the stability of SNIP1 (Fig. 4C). Moreover, *MKRN1* overexpression diminished the stability of SNIP1 (Fig. 4D). Interestingly, CHX treatment destabilised MKRN1, lending credence to earlier research indicating MKRN1 is destroyed via self-ubiquitination [11, 14]. Although *MKRN1* overexpression caused SNIP1 protein degradation, *MKRN1* expression did not inhibit *SNIP1* mRNA expression (Fig. 4E–F), suggesting that the influence of *MKRN1* on SNIP1 level occurred post-transcriptionally. Since MKRN1 is an E3 ubiquitin ligase, to investigate the mechanism of MKRN1-induced SNIP1 degradation in-depth, we used the proteasome inhibitor MG132 to investigate whether SNIP1 degradation was dependent on ubiquitination and the 26S proteasome. MG132 reversed the *MKRN1*-mediated degradation of SNIP1, indicating that *MKRN1* degrades SNIP1 through the proteasome pathway (Fig. 4G). Next, ubiquitination analysis showed that *MKRN1* knockdown decreased the SNIP1 ubiquitination level (Fig. 4H), whereas *MKRN1* overexpression increased the ubiquitination level (Fig. 4I). Ubiquitination analysis after MG132 treatment showed that *MKRN1* induces ubiquitination of SNIP1 (Fig. 4J). These results suggest that SNIP1 is a ubiquitinated substrate of MKRN1.

The RING structural domain with ubiquitin ligase activity is essential to the RING structural domain family, of which MKRN1 is a member. Its structural domain pattern is shown in Fig. 4K, with amino acid residues 281–334 at the N-terminal end forming the RING structural domain. A mutation at position 307, from histidine to glutamic acid (H307E), in the ring finger structural domain of MKRN1 results in loss of its E3 ligase activity [11]. Therefore, we constructed an E3 ligase mutant H307E of *MKRN1* (Fig. 4L). Although the MKRN1 (H307E) mutant could bind to SNIP1 (Fig. 4M), it did not induce SNIP1 degradation (Fig. 4N), and there were no large differences in endogenous SNIP1 levels as exogenous MKRN1 (H307E) was gradually increased (Fig. 4O). Meanwhile, CHX experiments also showed that the ability to induce SNIP1 instability was blocked after *MKRN1* mutation (Fig. 4P). We used ubiquitination analysis to validate the failure of *MKRN1* (H307E) to generate SNIP1 ubiquitination (Fig. 4Q). *MKRN1* (H307E) does not induce SNIP1 ubiquitination and degradation, demonstrating the requirement of its E3 ligase activity in mediating SNIP1 degradation. Our findings suggest that MKRN1 is a new E3 ligase of SNIP1.

### *MKRN1* promotes CRC cell migration by inhibiting SNIP1

**Fig. 5:**
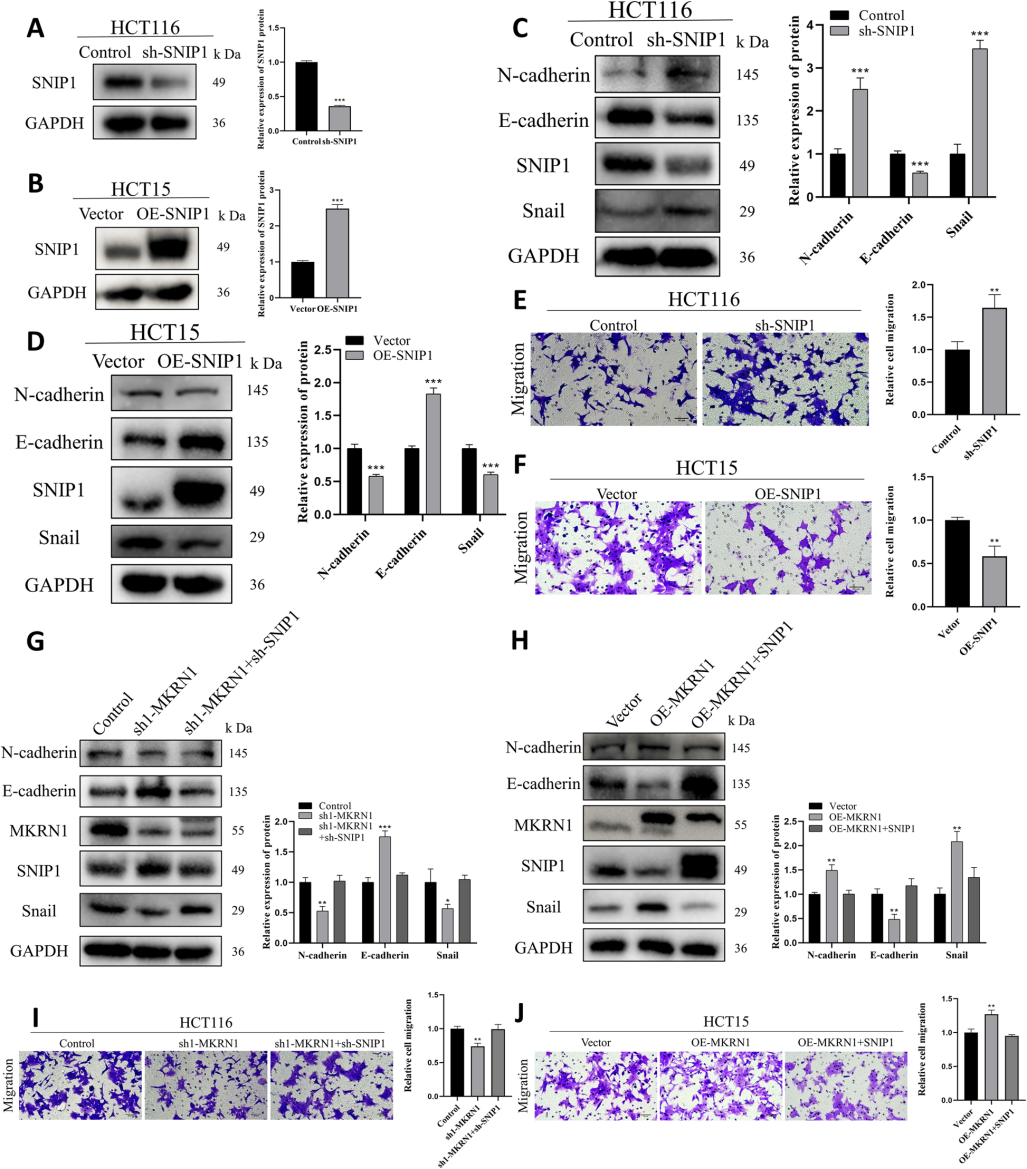
MKRN1 induces EMT in CRC cells by degrading SNIP1 protein. **A**, **B** WB was used to determine the transfection rate of *SNIP1* in HCT116 and HCT15 cells. **C**, **D** WB was used to measure expression levels of epithelial and mesenchymal markers following *SNIP1* knockdown and overexpression. **E**, **F** Transwell assays for migration and invasion of *SNIP1* knockdown and overexpressing cells (Scale bar: 50 µm). **G** WB was used to determine the level of major EMT proteins in HCT116 cells co-transfected with control, sh1-*MKRN1*, and sh-*SNIP1*. **H** WB analysis of the level of major EMT proteins in HCT15 cells co-transfected with vector, OE-*MKRN1*, and OE-*SNIP1*. **I** Transwell assay of the migration ability of HCT116 cells after co-transfection with control, sh1-*MKRN1*, and sh-*SNIP1* (Scale bar: 50 µm). **J** Transwell assay of HCT15 cells co-transfected with vector, OE-*MKRN1*, and OE-*SNIP1* for cell migration (scale bar: 50 µm). * *P* < 0.05, ** *P* < 0.01, *** *P* < 0.001

Downregulation of *SNIP1* reduced the expression of epithelial markers in multiple CRC cells [21]. To determine if *SNIP1* has a function in the suppression of CRC metastases, we silenced and overexpressed *SNIP1* in CRC cells (Fig. 5A, B). WB results showed that *SNIP1* knockdown in HCT116 cells increased N-cadherin and Snail expression and decreased the expression of E-cadherin, promoting EMT in CRC cells (Fig. 5C). *SNIP1* overexpression in HCT15 cells reduced N-cadherin and Snail levels, and enhanced expression of E-cadherin, inhibiting EMT in CRC cells (Fig. 5D). Transwell assays revealed that *SNIP1* knockdown enhanced HCT116 cell migratory capacity (Fig. 5E), whereas *SNIP1* overexpression reduced the migratory capacity of HCT15 cells (Fig. 5F). In addition, GSEA showed that SNIP1 was negatively correlated with the EMT pathway (Supplementary Fig. 1). These results demonstrate the ability of *SNIP1* to inhibit EMT and CRC cell migration.

We used a rescue experiment to evaluate whether *MKRN1* promotes CRC cell migration by *SNIP1*. Immunoblotting showed that silencing of *SNIP1* restored the EMT process in *MKRN1*-depleted cells (Fig. 5G). *SNIP1* overexpression reversed EMT activation in HCT15 cells induced by OE-*MKRN1* (Fig. 5H). Functional tests showed that *SNIP1* inhibition in HCT116 sh1-*MKRN1* cells significantly restored the migratory capacity of CRC cells (Fig. 5I). In contrast, *SNIP1* overexpression in HCT15 OE-*MKRN1* cells significantly inhibited the migratory ability of CRC cells (Fig. 5J). Our data indicate that *MKRN1* induces EMT in CRC cells via downregulation of SNIP1 expression.

### *MKRN1* facilitates EMT in CRC cells by stimulating the TGF-β signalling pathway via SNIP1 protein degradation

**Fig. 6:**
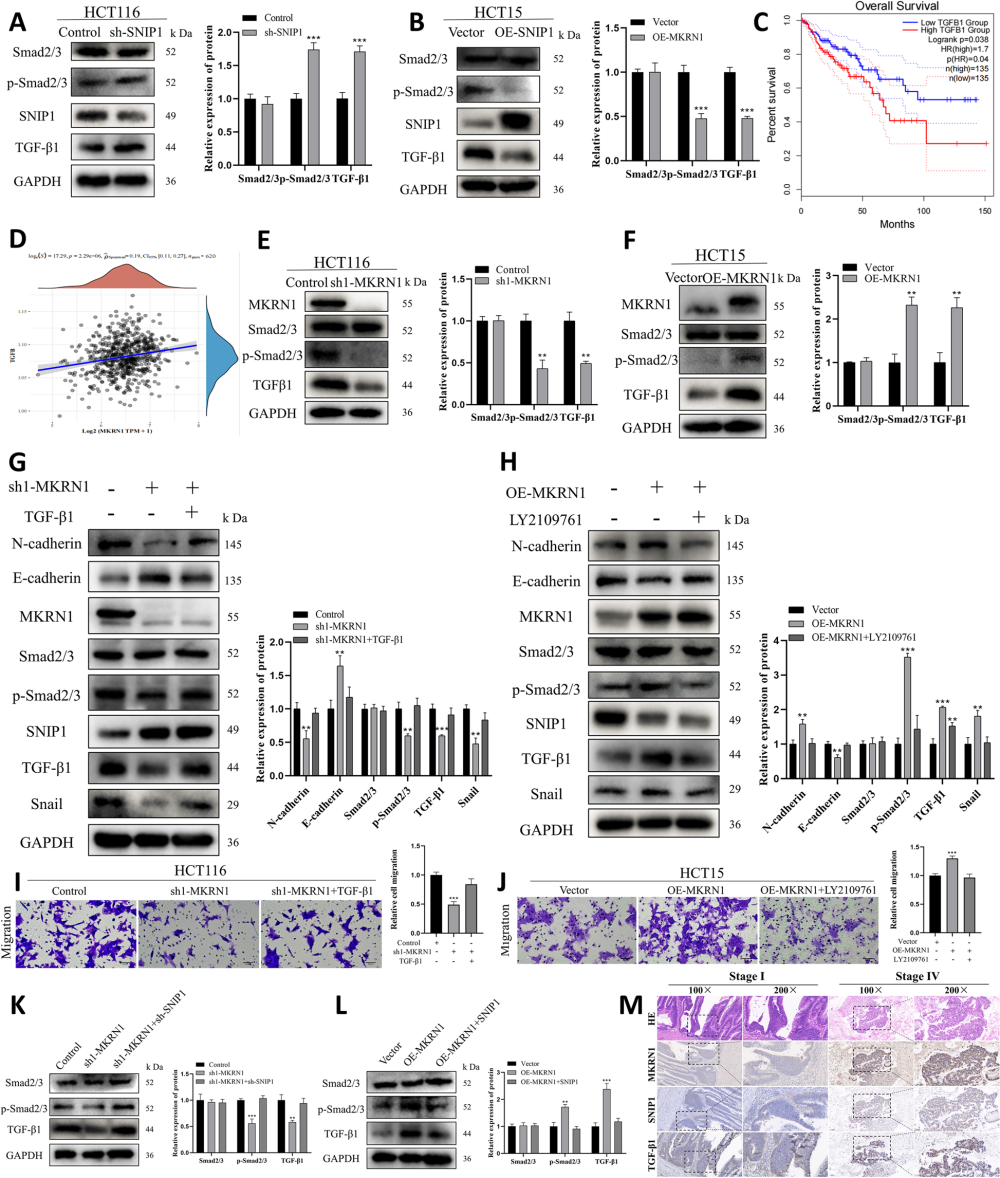
High MKRN1 levels promote the TGF-β signalling via SNIP1 ubiquitination and degradation. **A**, **B** WB was used to detect the effect of SNIP1 on the TGF-β pathway. **C***TGF-β1* expression was shown to be inversely associated to the prognosis of CRC patients. **D** Spearman correlation analysis between *MKRN1* and the TGF-β pathway. **E**, **F** WB detection of TGF-β pathway-related factors after *MKRN1* knockdown and overexpression. **G** TGF-β1 treatment reversed the inhibition of EMT and TGF-β signalling-related molecules induced by *MKRN1* knockdown. **H** LY2109761 treatment reversed the activation of EMT and TGF-β signalling-related molecules induced by *MKRN1* overexpression. **I** TGF-β1 treatment reversed the impaired migratory capacity of HCT116 cells caused by *MKRN1* knockdown (Scale bar: 50 µm). **J** LY2109761 treatment reversed the increased migratory capacity of HCT15 cells caused by *MKRN1* overexpression (Scale bar: 50 µm). **K** Expression of major TGF-β signalling markers after co-transfection of HCT116 cells with Control, sh1-*MKRN1*, and sh-*SNIP1*. **L** Expression of major TGF-β signalling markers after HCT15 cell co-transfection with vector, OE-*MKRN1*, and OE-*SNIP1*. **M** Representative graphs of MKRN1, SNIP1, and TGF-β1 expression in CRC detected by immunohistochemistry (scale bar: 100 µm; scale bar: 50 µm). ** *P* < 0.01, *** *P* < 0.001

SNIP1 is a nuclear protein that was cloned and identified because of its capacity to suppress the TGF-signalling pathway [19]. We explored whether SNIP1 affects the TGF-β pathway in CRC cells. WB findings revealed that *SNIP1* knockdown activated the TGF-β pathway and that *SNIP1* overexpression inhibited the TGF-β pathway (Fig. 6A, B). Furthermore, we performed single-gene GSEA of the dataset, which revealed an unknown relationship between SNIP1 and the TGF-β pathway in CRC; upon further investigation, we found that SNIP1 showed a negative association with the TGF-β pathway in testicular cancer (Supplementary Fig. 2). Overall, these finding support a role for a connection between SNIP1 and the TGF-β pathway in CRC. Meanwhile, Kaplan–Meier evaluation revealed that increased *TGF-β1* expression was associated with poor survival in patients with CRC (Fig. 6C).

To examine the relationship between *MKRN1* and the TGF-β signalling, we used RNA-Seq data and clinical information for CRC from the TCGA database. Spearman correlation analysis revealed a positive relationship between *MKRN1* and the TGF-β pathway (Fig. 6D). Immunoblotting showed that *MKRN1* knockdown and overexpression had opposite effects on TGF-β, inhibiting and activating the pathway, respectively (Fig. 6E, F), suggesting that high *MKRN1* levels may facilitate CRC progression through TGF-β signalling.

Since the TGF-β pathway plays a vital function in EMT induction, a critical step in tumour invasion and metastasis, we further determined whether *MKRN1* affects EMT in CRC cells via TGF-β signalling. Treatment with TGF-β1 (20 ng/mL, 4 h) rescued the EMT capacity of HCT116 sh1-*MKRN1* cells (Fig. 6G), and treatment with the TGF-β pathway inhibitor LY2109761 (10 μM, 2 h) rescued the EMT capacity of HCT15 OE-*MKRN1* cells (Fig. 6H). Additionally, we performed a functional assay using the transwell assay and showed that TGF-β1 treatment (10 ng/mL) substantially enhanced the migration ability of HCT116 sh1-*MKRN1* cells (Fig. 6I), while treatment with LY2109761 significantly reduced the migratory capacity of HCT15 OE-*MKRN1* cells (Fig. 6J). Furthermore, GSEA showed that *MKRN1* positively correlated with the TGF-β pathway (Supplementary Fig. 3A) and with the TGF-β-mediated EMT signalling pathway (Supplementary Fig. 3B). These results suggest that *MKRN1* promotes CRC cell migration through TGF-β signalling.

Furthermore, to assess whether high *MKRN1* expression promoting TGF-β signalling is associated with SNIP1 degradation, we introduced sh-*SNIP1* in HCT116 sh1-*MKRN1* cells. WB showed that the interference of *SNIP1* reversed the reduction in p-Smad2/3 and TGF-β1 levels in *MKRN1*-deficient cells. OE-*SNIP1* was introduced in HCT15 OE-*MKRN1* cells, which showed that *SNIP1* overexpression reduced the levels of p-Smad2/3 and TGF-β1 in *MKRN1* overexpressing cells (Fig. 6K, L).

We used IHC to validate the MKRN1, SNIP1, and TGF-β1 expression in clinical CRC specimens to confirm our previous study. In patients with high MKRN1 expression, SNIP1 expression was low, whereas TGF-β1 expression was strong (Fig. 6M). Our findings are consistent with the idea that *MKRN1* induces EMT in CRC cells by activating the TGF-β1 signalling pathway through SNIP1 degradation.

### *MKRN1* promotes tumour proliferation and metastasis in mice

**Fig. 7:**
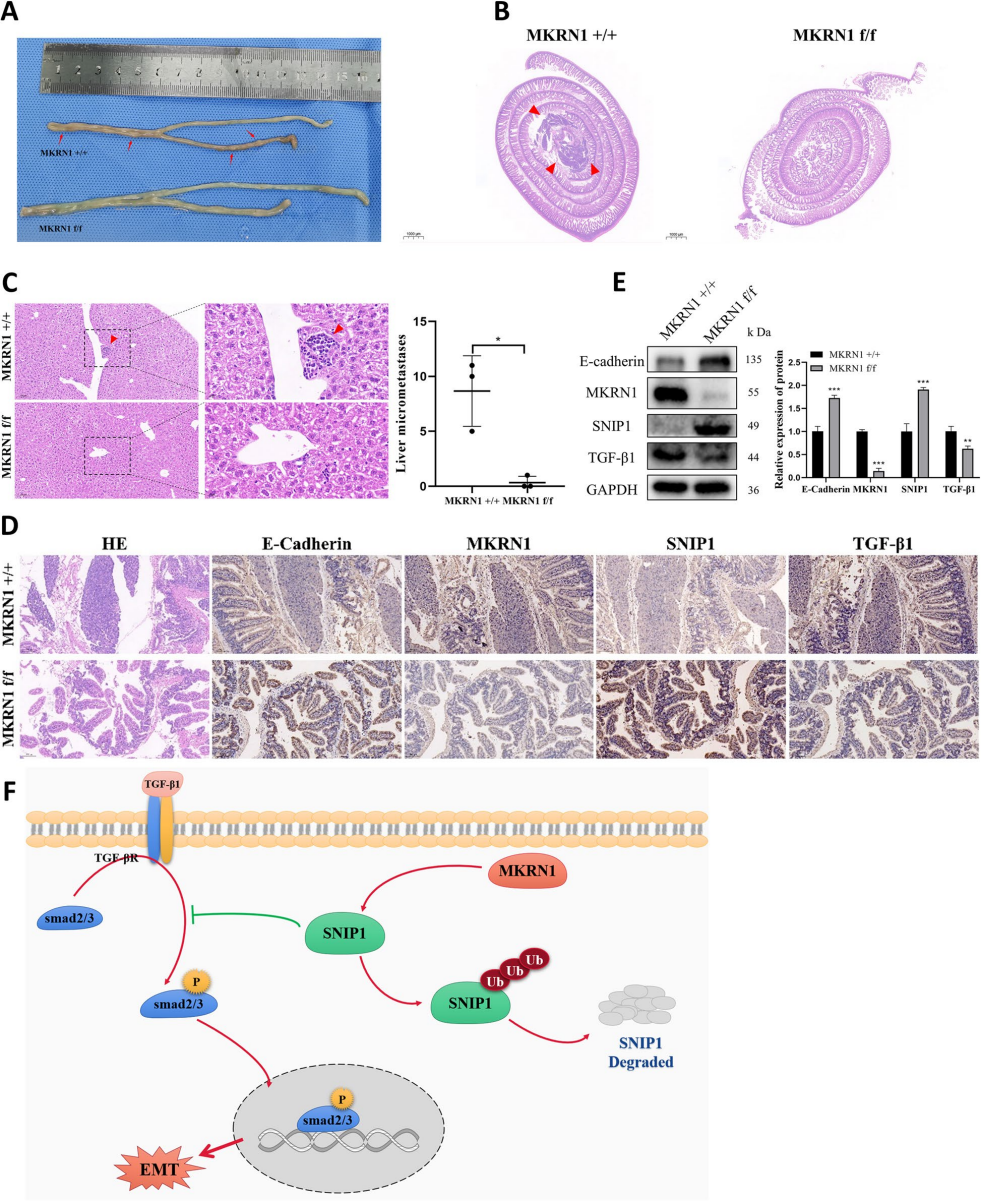
MKRN1 promotes tumour proliferation and metastasis in vivo. **A** Comparative graph showing the number of intestinal lesions in the *MKRN1* [+ / +] and *MKRN1* [f/f] groups. **B** Haematoxylin–eosin (H&E) staining of the intestine of both groups of mice (scale bar: 100 μm). **C** H&E staining of the liver in the two groups of mice (scale bar: 100 µm; scale bar: 20 µm). **D** IHC staining for E-cadherin, MKRN1, SNIP1, and TGF-β1 in the intestinal tissues of the two groups of mice (scale bar: 100 µm). **E** Western blotting analysis of E-cadherin, MKRN1, SNIP1, and TGF-β1 protein expression in intestinal tissues of the two groups of mice. **F ***MKRN1* facilitates the TGF-β signalling via ubiquitination and degradation of SNIP1, thereby promoting EMT in CRC cells. * *P* < 0.05, ** *P* < 0.01, *** *P* < 0.001

Apc mutations can cause CRC [26–28]. To investigate MKRN1's influence on CRC development in vivo, we constructed Apc [MU +] *MKRN1* CKO mice (Apc [MU/ +], Mkrn1 [+ / + , pVillin-Cre], Apc [MU/ +], and Mkrn1 [flox/flox, pVillin-Cre]). Mice were anaesthetised and euthanised at 33 weeks. Intestinal lesions were considerably reduced in the knockout *MKRN1* group compared to the control group (Fig. 7A and Supplementary Fig. 4A). Haematoxylin–eosin (H&E) staining revealed substantially fewer intestinal lesions and microscopic liver metastases in the knockout *MKRN1* group than in the control group (Fig. 7B, C). IHC analysis showed higher E-cadherin and SNIP1 levels and reduced TGF-β1 expression in the knockout *MKRN1* group than in the control group (Fig. 7D). WB analysis showed that E-cadherin and SNIP1 protein expression was increased, and that TGF-β1 expression was decreased in the intestinal cells of MKRN1 knockout mice relative to the control group (Fig. 7E), consistent with in vitro experiments. Notably, at 36 weeks of growth, a tumour was found in the left lower limb of one mouse in the *MKRN1*[+ / +] group (Supplementary Fig. 4B). Tumour tissue was used for IHC analysis, which revealed high MKRN1 and TGF-β1 expression and low SNIP1 and E-cadherin expression in the cancer cells (Supplementary Fig. 4C). Our findings suggested that MKRN1 has a role in tumorigenesis and metastasis in vivo.

These results suggest that MKRN1 promotes CRC cell invasion and EMT via ubiquitination and degradation of SNIP1, thus eliminating TGF-β signalling inhibition (Fig. 7F).

## Discussion

CRC is the most widespread gastrointestinal tract cancer [1], and metastasis is the leading cause of cancer mortality [29]. Therefore, reducing the incidence of metastases improves the lives and prognosis of patients with CRC [30]. EMT is an essential biological mechanism that is used by malignant cells of epithelial origin to migrate and invade [31]. The EMT pathway is gaining popularity as a potential cancer target for therapeutic intervention to prevent the spread of tumour cells in patients at an early stage of the disease or to eliminate current metastatic cells in patients with advanced diseases [32]. Therefore, searching for new EMT markers is critical to improving the survivability of patients with CRC.

In the current investigation, MKRN1 was discovered to be substantially expressed in CRC and that high *MKRN1* promoted CRC progression through EMT induction, a function mainly achieved through *MKRN1* ubiquitination and degradation of SNIP1, thereby activating the TGF-β pathway. *MKRN1* was discovered as a novel oncogenic factor that induces CRC progression.

MKRN1 is a member of the RNF family of proteins, also known as RNF61. At their N-terminus, RING finger proteins have a RING structural domain that interacts with ubiquitin and transports it to the protein substrate. RNF proteins play a function in the growth of various cancers [33]. For example, *RNF126* is linked to early breast cancer metastasis and promotes breast cancer cell proliferation and development [34]. High *RNF13* expression boosts the invasive ability of pancreatic tumours by enhancing matrix metalloproteinase-9 expression [35]. In addition, *RNF183* promotes the transition from inflammation to malignancy by stimulating the NF-κB–IL-8 axis in CRC [36]. MKRN1 directly interacts with and ubiquitinates Apc, thereby accelerating its proteasomal degradation and positively regulating Wnt/β-catenin-mediated proliferation and metastasis of cancer cells [37].

Here, we provide sufficient evidence that *MKRN1* promotes CRC progression. First, *MKRN1* is extensively expressed in CRC and positively correlates with the clinical TNM stage, and patients with CRC with high *MKRN1* expression have a worse prognosis. Second, in vitro studies have shown that *MKRN1*-positive CRC cells exhibit a mesenchymal phenotype, which promotes their proliferation, migration, and invasiveness, mirroring EMT processes in CRC cells. Finally, in vivo experiments demonstrated that mice in the *MKRN1* knockout group had substantially fewer intestinal lesions and microfoci of liver metastases than controls. These results reveal the oncogenic potential of *MKRN1* in CRC.

The ubiquitin–proteasome system is the major mechanism of protein degradation in the cytoplasm and nucleus, and is critical for maintaining protein homeostasis [38]. E3 ubiquitin ligase is a key element of the ubiquitin–proteasome system and is implicated in cancer formation by binding to and triggering the degradation of target proteins [39]. As E3, MKRN1 physically interacts with several target proteins for ubiquitination and proteasomal degradation.

Our study showed that MKRN1 co-localises with SNIP1. Co-IP results showed that both endo- and exo-derived MKRN1 interacted with SNIP1. *MKRN1* destabilises SNIP1 and mediates SNIP1 degradation by the ubiquitin–proteasome pathway, suggesting that SNIP1 is a novel substrate for MKRN1 ubiquitination. Notably, although mutant MKRN1 (H307E) is defective in E3 ligase activity, it can bind to SNIP1, but does not induce SNIP1 degradation and ubiquitination, suggesting that this site may not be critical for MKRN1 binding to SNIP1 but is essential for the MKRN1 ubiquitination of SNIP1. Thus, the study of synthetic compounds targeting the H307E mutation site of MKRN1 for CRC inhibition deserves further exploration.

Notably, SNIP1 can be SUMO-modified at its K5, K30, and K108 sites. SNIP1 SUMOylation interferes with Smad complex formation and leads to increased expression of TGFβ target genes, thereby promoting TGFβ signalling-mediated cell migration and infiltration [17]. Accordingly, we verified the role of *SNIP1* in CRC cells and confirmed by WB and transwell assays that silencing *SNIP1* promotes EMT and migration in CRC cells. Hence, we hypothesized that *MKRN1* would influence EMT process in CRC cells by degrading SNIP1. Consistent with this speculation, rescue experiments demonstrated that silencing of *SNIP1* reversed the inhibition of EMT markers by sh1-*MKRN1*. This study reported for the first time that the expression of MKRN1 and SNIP1 in CRC were negatively correlated, confirming that SNIP1 can act as a tumour suppressor and can be modified by MKRN1 ubiquitination at the protein level. Moreover, *MKRN1* promoted metastasis and EMT in CRC cells by ubiquitinating SNIP1. Our results are consistent with literature reporting that *SNIP1* can inhibit epithelial markers [21, 40]. Our results provide novel insight into the inactivation of this tumour suppressor.

TGF-β belongs to the TGF superfamily and provides tissue-specific control over proliferation and cell- and tissue-specific motility [41]. The TGF-β signalling pathway can produce pro-infiltrative and pro-metastatic effects through EMT induction in various cancers [42]. TGF-β is also associated with CRC [43] and promotes EMT in this cancer [44]. Preoperative TGF-β levels in CRC predict liver metastasis following therapeutic resection, implying that TGF-β-positive CRC cells may have the biological capability for liver metastasis [45].

Here, we confirmed that *MKRN1* was positively correlated with the TGF-β pathway by bioinformatics analysis and WB. In addition, we demonstrated that high expression of *MKRN1* activates TGF-β signalling and thus promotes EMT in CRC cells using TGF-β signalling inducers and inhibitors. However, whether targeted treatment of CRC using TGF-β pathway inhibitors can reduce the incidence of tumour metastasis requires further investigation.

SNIP1 interacts with Smad4 to suppress recruitment to the co-transcription factor CBP/p300, inhibiting TGF-β pathway activation [15]. However, the link between *SNIP1* and the TGF-β signalling in CRC was unclear. Accordingly, we validated the connection between *SNIP1* and the TGF-β signalling in CRC cells and showed that silencing *SNIP1* inhibited TGF-β signalling, and *SNIP1* overexpression promoted TGF-β signalling. Furthermore, we demonstrated through rescue experiments that silencing *SNIP1* reversed the inhibition of TGF-β signalling pathway-related markers by sh1-*MKRN1*.

Recent studies in tumour metastasis have found that at the transcriptional level, deletion of E-cadherin is associated with the upregulation of genes in the TGFβ transduction pathway [46]. We performed IHC staining using tissue sections from CKO mice and clinical patients. The results suggested that the MKRN1 high-expression group had lesser SNIP1 and E-cadherin levels but increased TGF-β1 expression. By combining in vitro and in vivo studies, this work demonstrated that *MKRN1* overexpression plays an essential role in CRC development by promoting TGF-β signalling via SNIP1 ubiquitination and degradation.

However, there are still some limitations to this study. The specific binding regions of MKRN1 and SNIP1 and the lysine site of MKRN1 targeting SNIP1 for ubiquitination modification have not been confirmed. In future studies, we will identify binding areas and specific ubiquitination sites.

## Conclusions

Overall, we present a dependable factor that may be employed as a possible diagnostic biomarker, prognostic indicator, and therapy for CRC. This research shows that *MKRN1* is abundantly expressed in CRC and activates TGF-β signalling through SNIP1 ubiquitination and degradation, thereby promoting EMT and migration of CRC cells. Accordingly, *MKRN1* could be a promising biomarker for predicting CRC metastasis, and the MKRN1/SNIP1/TGF-β axis may provide a new target for developing anti-metastatic drugs for CRC.

## Supplementary Information

**Additional file 1.** IHC.

**Additional file 2.** Primary antibody information.

**Additional file 3.** PCR Primer List.

**Additional file 4.** Quantitative proteomics.

**Additional file 5: Supplementary Figure S1. **High SNIP1 expression inhibits the EMT pathway. TCGA–TGCT dataset was downloaded, SNIP1 was divided into high and low expression groups, and the differentially expressed genes in these groups were scored for enrichment using GSEA. The vertical coordinate of the graph is the enrichment score, and the horizontal coordinate is the number of times the samples were scored for the calculation; on the left is the SNIP1 high-expression group, and on the right is the SNIP1 low-expression group; the peak of this enrichment is mainly enriched in the low-expression group. The results satisfy *|NES|*> 1, *P* < 0.05, and *FDR* < 0.25, indicating that the results are meaningful and significant. **Supplementary Figure S2.** Low SNIP1 expression can activate the TGF-β pathway. TCGA–TGCT dataset was downloaded, SNIP1 was divided into high and low expression groups, and then the differentially expressed genes in these groups were scored for enrichment using GSEA. The vertical coordinate of the graph is the enrichment score, and the horizontal coordinate is the number of times the samples were scored for the calculation; on the left is the SNIP1 high-expression group, and on the right is the SNIP1 low-expression group; the peak of this enrichment is mainly enriched in the low-expression group. The results satisfy *|NES|* > 1, *P*< 0.05, and *FDR* < 0.25, indicating that the results are meaningful and significant. **Supplementary Figure S3. **MKRN1 positively correlates with the TGF-β-mediated EMT signalling pathway. A–B)TCGA–COAD dataset was downloaded, MKRN1 was divided into high and low expression groups, and then the differentially expressed genes in these groups were scored for enrichment using GSEA. The vertical coordinate of the graph is the enrichment score, and the horizontal coordinate is the number of times the samples were scored for the calculation; on the left is the MKRN1 high-expression group, and on the right is the MKRN1 low-expression group. The results satisfy *|NES|* > 1, *P* < 0.05, and *FDR* < 0.25, indicating that the results are meaningful and significant. **Supplementary Figure S4.** In vivo, MKRN1 promotes proliferation and metastasis of colorectal cancer. A) Photograph of intestinal lesions in the MKRN1[+/+] group of mice. B) Tumours in the left lower limb of a mouse in the MKRN1[+/+] group. C) HE and immunohistochemical staining for the expression of MKRN1, SNIP1, TGF-β1, and E-Cadherin in tumour tissues (Scale bar: 50 µm).

## Data Availability

The datasets generated and/or analysed during the current study are available from the supplementary information or the corresponding author upon reasonable request.

## Abbreviations

CRC: Colorectal cancer
EMT: Epithelial-mesenchymal transition
hTERT: Human telomerase reverse transcriptase
FADD: Fas-associated protein with data domain
GSEA: Gene set enrichment analysis
H&E: Haematoxylin–eosin
IHC: Immunohistochemistry
IP: Immunoprecipitation
MKRN1: Makorin ring finger protein 1
PTEN: Phosphatase and tensin homologue
SNIP1: Smad nuclear-interacting protein 1
TCGA: The Cancer Genome Atlas databases
TGF-β: Transforming growth factor-β
TNM: Tumour, nodes, and metastases
WB: Western blotting
