# Correction: MKRN1 promotes colorectal cancer metastasis by activating the TGF-β signalling pathway through SNIP1 protein degradation

**DOI:** 10.1186/s13046-023-02825-8

**Published:** 2023-09-15

**Authors:** Yi Zhang, Qinshan Li, Honglin Liu, Hongting Tang, Hanlin Yang, Daoqiu Wu, Yuying Huang, Licheng Li, Lihong Liu, Mengxing Li

**Affiliations:** 1 Guizhou Prenatal Diagnosis Center, Afliated Hospital of Guizhou Medical University, Guiyang, Guizhou, 550004 People’s Republic of China; 2 Department of Clinical Biochemistry, School of Medical Laboratory ScienceGuizhou Medi cal University, Guizhou, Guiyang, 550004 People’s Republic of China; 3 Institute of Clinical Medical Sciences, China-Japan Friendship Hospital, Beijing, 100000 People’s Republic of China; 4 Clinical Medical College, Guizhou Medical Uni versity, Guizhou, Guiyang, 550004 People’s Republic of China; 5 Department of HematologyGuizhou Province Laboratory of Hematopoietic Stem Cell Trans plantation Centre, Afliated Hospital of Guizhou Medical University, Guizhou Province Institute of Hematology, Guizhou, Guiyang, People’s Republic of China; 6 Department of Pharmacy, China-Japan Friendship Hospital, Beijing, 100029 People’s Republic of China; 7 Department of Pathophysiology, Guizhou Medical University, Guizhou, Guiyang, 550004 People’s Republic of China

**Correction**: ***J Exp Clin Cancer Res *42, 219 (2023)**

10.1186/s13046-023-02788-w

Following publication of the original article [1], minor error was identified in the second word of Keywords section. Letter “M” was omitted, the correct acronym should be ‘*MKRN1*’.

The correction does not affect the overall result or conclusion of the article. The original article has been corrected.
