# Correction: MKRN1 promotes colorectal cancer metastasis by activating the TGF-β signalling pathway through SNIP1 protein degradation

**DOI:** 10.1186/s13046-024-03265-8

**Published:** 2025-01-04

**Authors:** Yi Zhang, Qin-shan Li, Hong-lin Liu, Hong-ting Tang, Han-lin Yang, Dao-qiu Wu, Yu-ying Huang, Li-cheng Li, Li-hong Liu, Meng-xing Li

**Affiliations:** 1 Guizhou Prenatal Diagnosis Center, Affiliated Hospital of Guizhou Medical University, Guiyang, Guizhou 550004 People’s Republic of China; 2 Department of Clinical Biochemistry, School of Medical Laboratory Science, Guizhou Medical University, Guizhou Guiyang, 550004 People’s Republic of China; 3 Institute of Clinical Medical Sciences, China-Japan Friendship Hospital, Beijing, 100000 People’s Republic of China; 4 Clinical Medical College, Guizhou Medical University, Guizhou Guiyang, 550004 People’s Republic of China; 5 Department of HematologyGuizhou Province Laboratory of Hematopoietic Stem Cell Transplantation Centre, Affiliated Hospital of Guizhou Medical University, Guizhou Province Institute of Hematology, Guizhou Guiyang, People’s Republic of China; 6 Department of Pharmacy, China-Japan Friendship Hospital, Beijing, 100029 People’s Republic of China; 7 Department of Pathophysiology, Guizhou Medical University, Guizhou Guiyang, 550004 People’s Republic of China

**Correction: J Exp Clin Cancer Res42**, ** 219 (2023)**

10.1186/s13046-023-02788-w

Following the publication of the original article [1], the authors identified an error in Fig. 7D. The image presented for the MKRN1 f/f group (IHC staining of TGF-β1) was inadvertently incorrect due to an oversight during figure preparation.

The correct figure is presented below:

**Correct Fig. 7D**

**Fig. 7:**
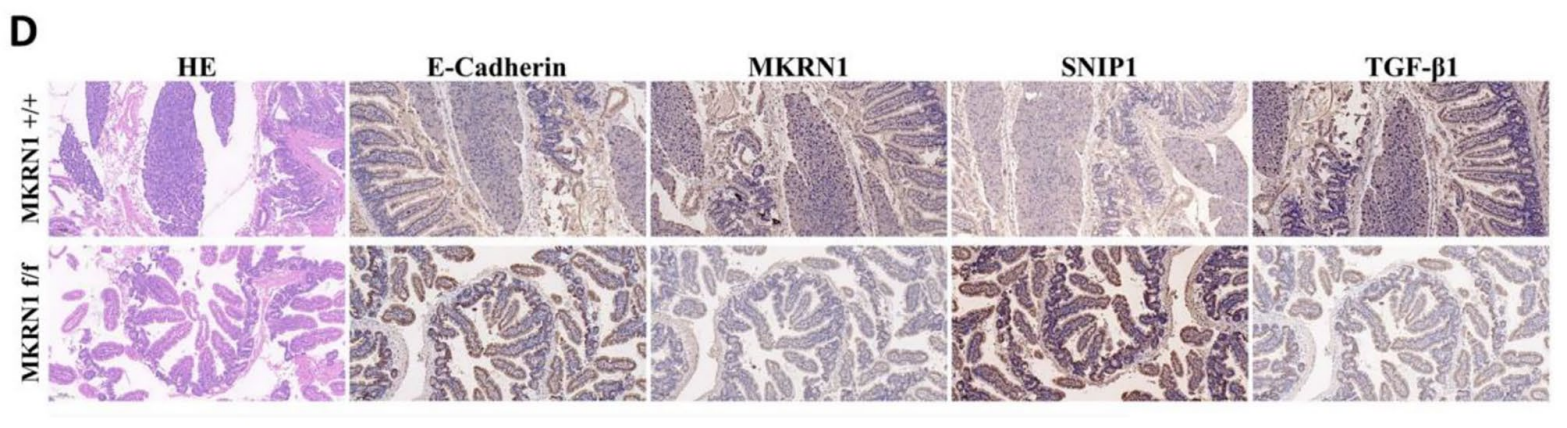
MKRN1 promotes tumour proliferation and metastasis in vivo. **A** Comparative graph showing the number of intestinal lesions in the *MKRN1* [+/+] and *MKRN1* [f/f ] groups. **B** Haematoxylin–eosin (H&E) staining of the intestine of both groups of mice (scale bar: 100 μm). **C** H&E staining of the liver in the two groups of mice (scale bar: 100 μm; scale bar: 20 μm). **D** IHC staining for Ecadherin, MKRN1, SNIP1, and TGFβ1 in the intestinal tissues of the two groups of mice (scale bar: 100 μm). **E** Western blotting analysis of Ecadherin, MKRN1, SNIP1, and TGFβ1 protein expression in intestinal tissues of the two groups of mice. **F** *MKRN1* facilitates the TGFβ signalling via ubiquitination and degradation of SNIP1, thereby promoting EMT in CRC cells. *P < 0.05, *P < 0.01, * *P* < 0.001

**Incorrect Fig. 7D**

**Fig. 7:**
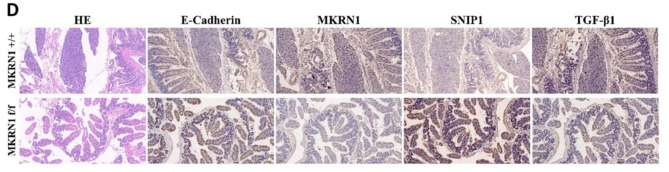
MKRN1 promotes tumour proliferation and metastasis in vivo. **A** Comparative graph showing the number of intestinal lesions in the *MKRN1* [+/+] and *MKRN1* [f/f ] groups. **B** Haematoxylin–eosin (H&E) staining of the intestine of both groups of mice (scale bar: 100 μm). **C** H&E staining of the liver in the two groups of mice (scale bar: 100 μm; scale bar: 20 μm). **D** IHC staining for Ecadherin, MKRN1, SNIP1, and TGFβ1 in the intestinal tissues of the two groups of mice (scale bar: 100 μm). **E** Western blotting analysis of Ecadherin, MKRN1, SNIP1, and TGFβ1 protein expression in intestinal tissues of the two groups of mice. **F** *MKRN1* facilitates the TGFβ signalling via ubiquitination and degradation of SNIP1, thereby promoting EMT in CRC cells. *P < 0.05, *P < 0.01, * *P* < 0.001

The correction does not compromise the validity of the conclusions and the overall content of the article. The original article [1] has been updated.
