# Supplementary material for: MKRN1 promotes colorectal cancer metastasis by activating the TGF-β signalling pathway through SNIP1 protein degradation
Source: J Exp Clin Cancer Res. 2023 Aug 24;42:219. doi: 10.1186/s13046-023-02788-w (PMC10464235; doi:10.1186/s13046-023-02788-w)
Supplement: Supplementary file 1 — Additional file 1. IHC. [file 13046_2023_2788_MOESM1_ESM.doc]

**IHC**

# Experimental Procedures

1. Paraffin slices dewaxed to water: sequentially put the slices into environmentally friendly dewaxing solution Ⅰ for 10min - environmentally friendly dewaxing solution Ⅱ for 10min - environmentally friendly dewaxing solution Ⅲ for 10min - anhydrous ethanol Ⅰ for 5min - anhydrous ethanol Ⅱ for 5min - anhydrous ethanol Ⅲ for 5min - distilled water wash.
2. Antigen repair: this procedure should prevent excessive evaporation of the buffer and never dry the slide. After natural cooling, the slide is placed in PBS (pH 7.4) on a decolorization shaker and washed 3 times for 5 min each. (The repair solution and repair conditions are determined according to the tissue)
3. Blocking of endogenous peroxidase: sections were placed in 3% hydrogen peroxide solution and incubated for 25 min at room temperature and protected from light. Slides were placed in PBS (pH 7.4) and washed 3 times for 5 min each time by shaking on a decolorization shaker.
4. Serum closure: add 3% BSA dropwise to the histochemical circle to cover the tissue uniformly and close it for 30 min at room temperature (rabbit serum for primary antibody of goat origin, BSA for other sources) Serum closure: add 3% BSA dropwise to the histochemical circle to cover the tissue uniformly and close it for 30 min at room temperature (rabbit serum for primary antibody of goat origin, BSA for other sources)
5. Add primary antibody: Gently shake off the blocking solution, add a drop of PBS to the section in a proportion to the primary antibody, and incubate the section flat in a wet box at 4°C overnight.
6. Addition of secondary antibody: slides were washed 3 times in PBS (pH 7.4) on a decolorization shaker with shaking for 5 min each time. sections were slightly shaken dry and the tissue was covered with a secondary antibody (HRP-labelled) of the appropriate species in a circle drop and incubated for 50 min at room temperature.
7. DAB color development: slides were placed in PBS (pH 7.4) on a decolorization shaker and washed 3 times for 5 min each. sections were slightly shaken dry and then freshly prepared DAB color development solution was added dropwise in a circle for a controlled time under the microscope, positive for brownish yellow, and the color development was terminated by rinsing the sections with tap water.
8. Re-staining of nuclei: Hematoxylin re-staining for about 3 min, tap water wash, hematoxylin differentiation solution for a few seconds, tap water rinse, hematoxylin blue return solution for blue return, running water rinse.
9. Water sealing: put the slices into 75% alcohol for 5min - 85% alcohol for 5min - anhydrous ethanol Ⅰ for 5min - anhydrous ethanol Ⅱ for 5min - n-butanol for 5min - xylene Ⅰ for 5min to dehydrate and be transparent, take the slices out of xylene to dry slightly and seal the slices with sealing glue.
10. Microscopic examination: placed under a white light microscope for interpretation of the results.
