# Supplementary material for: MKRN1 promotes colorectal cancer metastasis by activating the TGF-β signalling pathway through SNIP1 protein degradation
Source: J Exp Clin Cancer Res. 2023 Aug 24;42:219. doi: 10.1186/s13046-023-02788-w (PMC10464235; doi:10.1186/s13046-023-02788-w)
Supplement: Supplementary file 2 — Additional file 2. Primary antibody information. [file 13046_2023_2788_MOESM2_ESM.doc]

**Primary antibody information**

| Designation | Company |
| --- | --- |
| MKRN1 | Bethyl, USA |
| SNIP1 | Proteintech, China |
| E-cadherin | CST, USA |
| N-cadherin | CST, USA |
| Snail | CST, USA |
| Ub | Abcam, UK |
| TGF-β1 | Abcam, UK |
| Smad2/3 | Affinity, USA |
| p- Smad2/3 | Affinity, USA |
| GAPDH | CST, USA |
