# Supplementary material for: MKRN1 promotes colorectal cancer metastasis by activating the TGF-β signalling pathway through SNIP1 protein degradation
Source: J Exp Clin Cancer Res. 2023 Aug 24;42:219. doi: 10.1186/s13046-023-02788-w (PMC10464235; doi:10.1186/s13046-023-02788-w)
Supplement: Supplementary file 3 — Additional file 3. PCR Primer List. [file 13046_2023_2788_MOESM3_ESM.doc]

**PCR Primer List**

**Table.1 primer sequences**

| **Name** | **Sequence** （5′-3′） | | **Output length**（bp） |
| --- | --- | --- | --- |
| MKRN1  SNIP1 | Sense  Antisense  Sense  Antisense | GCATTGAGGCCCATGAGAAG  GTAGGTGTGGTTGCAGTTGG  GAAACCGAAGTCCTCACCAC  TTCCTGTTCTGATGGTTCCC | 152  107 |
| GAPDH | Sense  Antisense | GGAGCGAGATCCCTCCAAAAT  GGCTGTTGTCATACTTCTCATGG | 197 |

**Table.2 Three shRNA target sequences of MKRN1 gene**

| **Name** | **Sequence** |
| --- | --- |
| sh1 | 5′-TGC TGT TGA CAG TGA GCG CCC TGG TTA TCC CAT ATA ATA ATA GTG AAG CCA CAG ATG TAT TAT TAT ATG GGA TAA CCA GGT TGC CTA CTG CCT CGG A-3′ |
| sh2 | 5′-TGC TGT TGA CAG TGA GCG ACG GAA CTA TGT GAT CCC GGA ATA GTG AAG CCA CAG ATG TAT TCC GGG ATC ACA TAG TTC CGG TGC CTA CTG CCT CGG A-3′ |
| sh3 | 5′-TGC TGT TGA CAG TGA GCG CTG CTG TGT GGG ATA AAC AGT ATA GTG AAG CCA CAG ATG TAT ACT GTT TAT CCC ACA CAG CAA TGC CTA CTG CCT CGG A-3′ |
