# Supplementary material for: MKRN1 promotes colorectal cancer metastasis by activating the TGF-β signalling pathway through SNIP1 protein degradation
Source: J Exp Clin Cancer Res. 2023 Aug 24;42:219. doi: 10.1186/s13046-023-02788-w (PMC10464235; doi:10.1186/s13046-023-02788-w)
Supplement: Supplementary file 5 — Additional file 5: Supplementary Figure S1. High SNIP1 expression inhibits the EMT pathway. TCGA–TGCT dataset was downloaded, SNIP1 was divided into high and low expression groups, and the differentially expressed genes in these groups were scored for enrichment using GSEA. The vertical coordinate of the graph is the enrichment score, and the horizontal coordinate is the number of times the samples were scored for the calculation; on the left is the SNIP1 high-expression group, and on the right is the SNIP1 low-expression group; the peak of this enrichment is mainly enriched in the low-expression group. The results satisfy |NES|> 1, P < 0.05, and FDR < 0.25, indicating that the results are meaningful and significant. Supplementary Figure S2. Low SNIP1 expression can activate the TGF-β pathway. TCGA–TGCT dataset was downloaded, SNIP1 was divided into high and low expression groups, and then the differentially expressed genes in these groups were scored for enrichment using GSEA. The vertical coordinate of the graph is the enrichment score, and the horizontal coordinate is the number of times the samples were scored for the calculation; on the left is the SNIP1 high-expression group, and on the right is the SNIP1 low-expression group; the peak of this enrichment is mainly enriched in the low-expression group. The results satisfy |NES| > 1, P< 0.05, and FDR < 0.25, indicating that the results are meaningful and significant. Supplementary Figure S3. MKRN1 positively correlates with the TGF-β-mediated EMT signalling pathway. A–B)TCGA–COAD dataset was downloaded, MKRN1 was divided into high and low expression groups, and then the differentially expressed genes in these groups were scored for enrichment using GSEA. The vertical coordinate of the graph is the enrichment score, and the horizontal coordinate is the number of times the samples were scored for the calculation; on the left is the MKRN1 high-expression group, and on the right is the MKRN1 low-expression group. Th [file 13046_2023_2788_MOESM5_ESM.doc]

**Supplementary figures and legends**

**
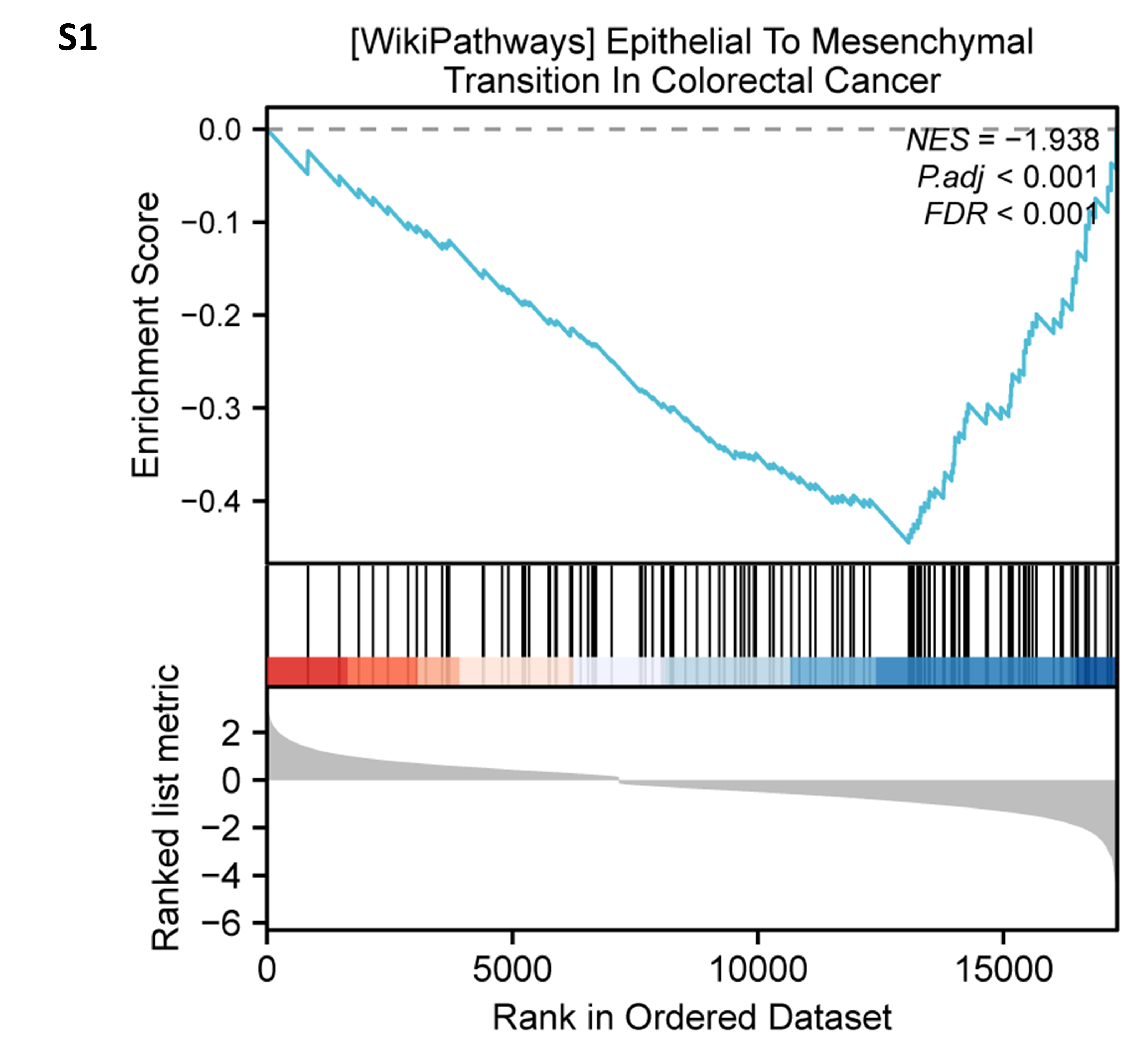
**

**Figure S1: High SNIP1 expression inhibits the EMT pathway** TCGA–TGCT dataset was downloaded, SNIP1 was divided into high and low expression groups, and the differentially expressed genes in these groups were scored for enrichment using GSEA. The vertical coordinate of the graph is the enrichment score, and the horizontal coordinate is the number of times the samples were scored for the calculation; on the left is the SNIP1 high-expression group, and on the right is the SNIP1 low-expression group; the peak of this enrichment is mainly enriched in the low-expression group. The results satisfy *|NES|* > 1, *P* < 0.05, and *FDR* < 0.25, indicating that the results are meaningful and significant.

**
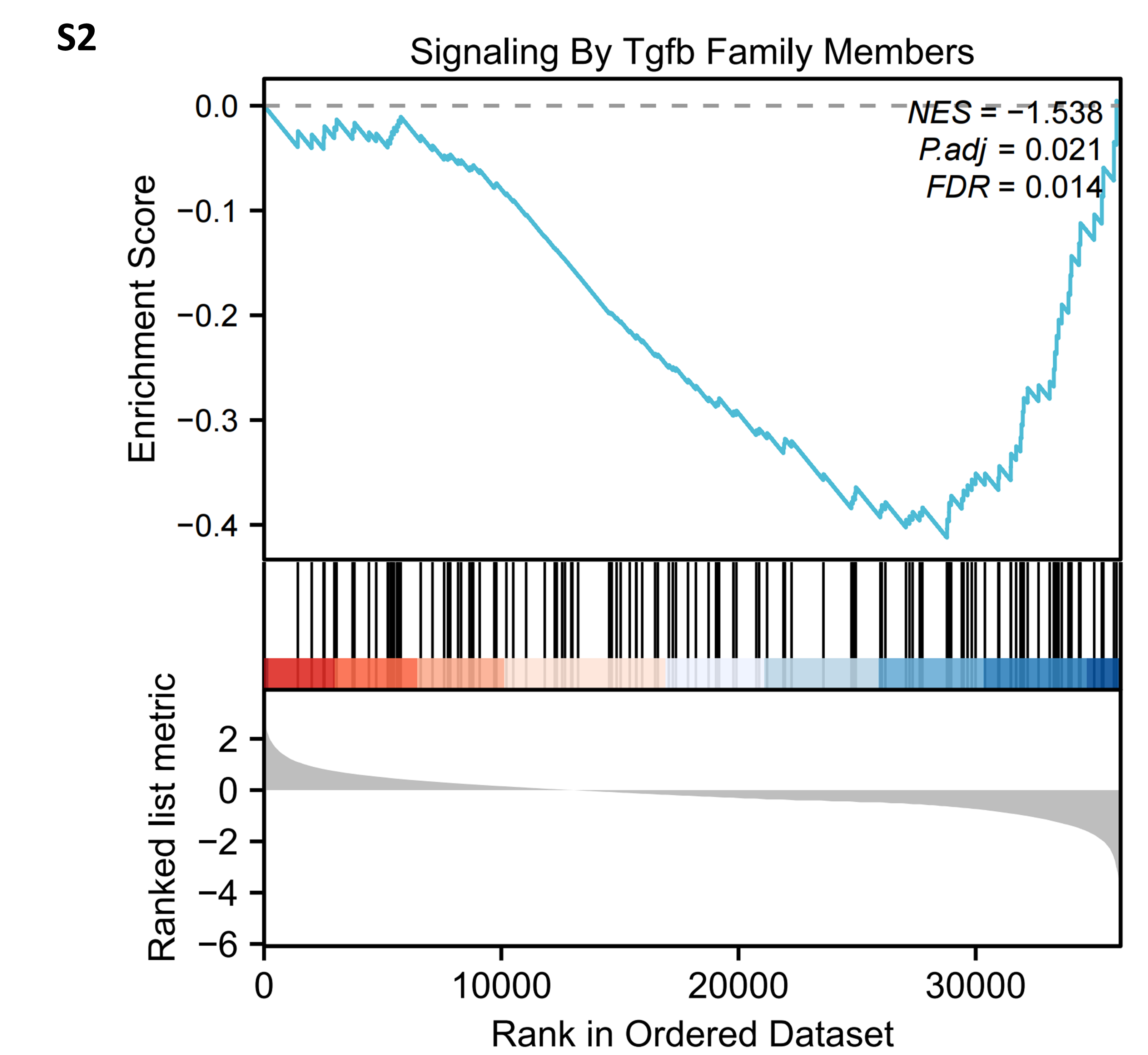
**

**Figure S2: Low SNIP1 expression can activate the TGF-β pathway** TCGA–TGCT dataset was downloaded, SNIP1 was divided into high and low expression groups, and then the differentially expressed genes in these groups were scored for enrichment using GSEA. The vertical coordinate of the graph is the enrichment score, and the horizontal coordinate is the number of times the samples were scored for the calculation; on the left is the SNIP1 high-expression group, and on the right is the SNIP1 low-expression group; the peak of this enrichment is mainly enriched in the low-expression group. The results satisfy *|NES|* > 1, *P* < 0.05, and *FDR* < 0.25, indicating that the results are meaningful and significant.


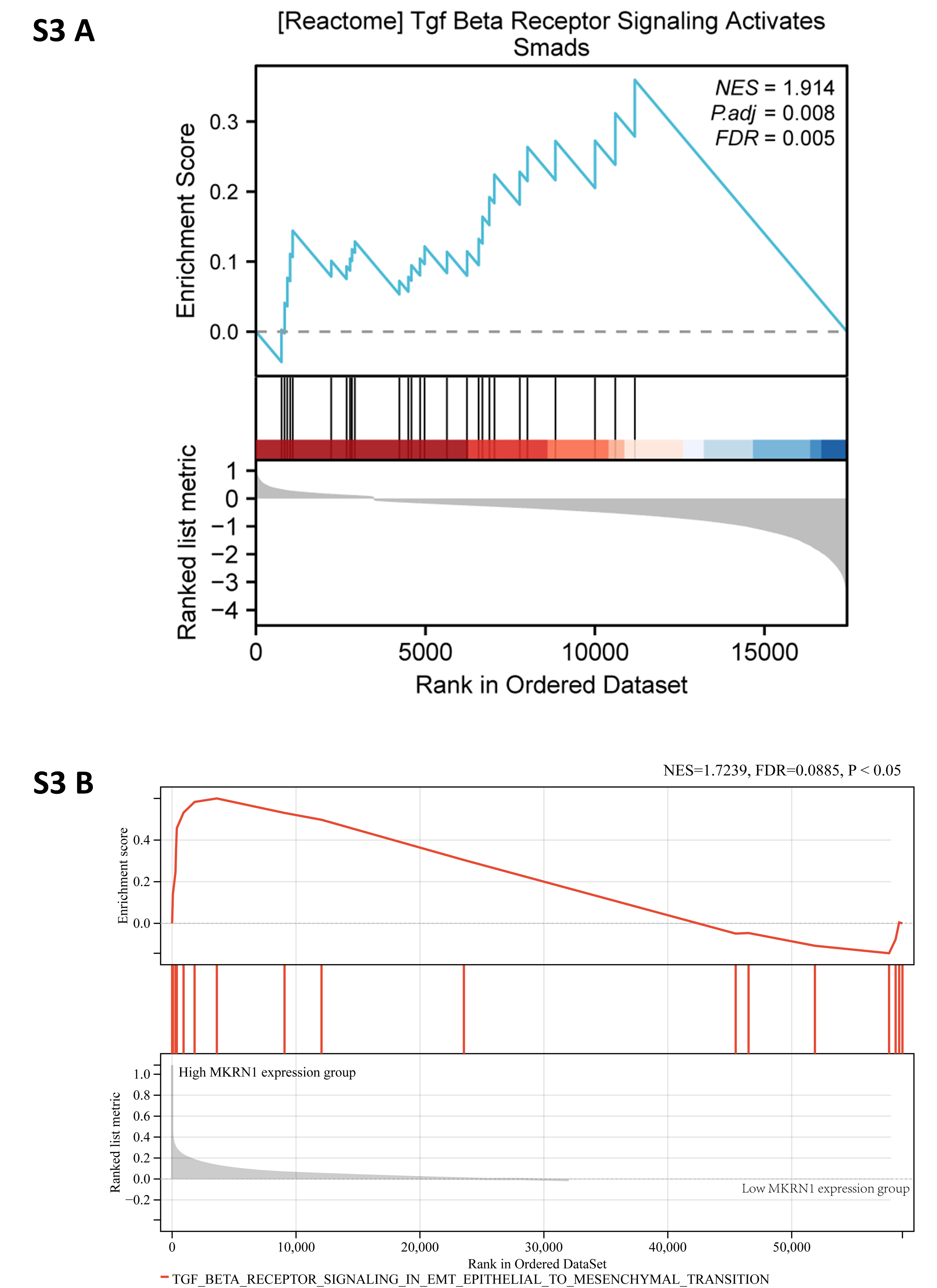


**Figure S3: MKRN1 positively correlates with the TGF-β-mediated EMT signalling pathway** A–B) TCGA–COAD dataset was downloaded, MKRN1 was divided into high and low expression groups, and then the differentially expressed genes in these groups were scored for enrichment using GSEA. The vertical coordinate of the graph is the enrichment score, and the horizontal coordinate is the number of times the samples were scored for the calculation; on the left is the MKRN1 high-expression group, and on the right is the MKRN1 low-expression group. The results satisfy *|NES|* > 1, *P* < 0.05, and *FDR* < 0.25, indicating that the results are meaningful and significant.


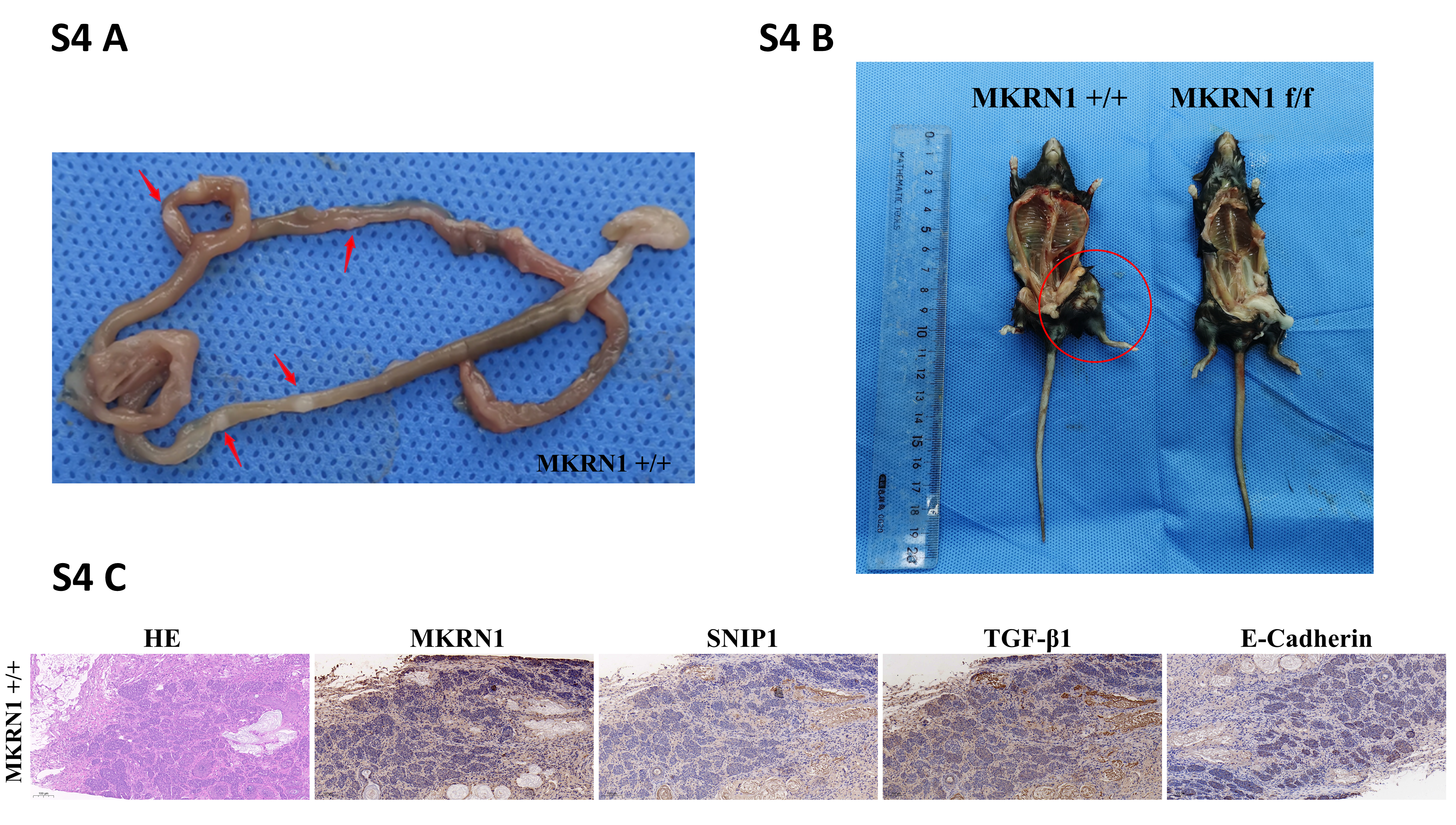


**Figure S4: In vivo, MKRN1 promotes proliferation and metastasis of colorectal cancer** A) Photograph of intestinal lesions in the MKRN1[+/+] group of mice. B) Tumours in the left lower limb of a mouse in the MKRN1[+/+] group. C) HE and immunohistochemical staining for the expression of MKRN1, SNIP1, TGF-β1, and E-Cadherin in tumour tissues (Scale bar: 50 µm).
